# The gut mycobiome of healthy mice is shaped by the environment and correlates with metabolic outcomes in response to diet

**DOI:** 10.1038/s42003-021-01820-z

**Published:** 2021-03-05

**Authors:** Tahliyah S. Mims, Qusai Al Abdallah, Justin D. Stewart, Sydney P. Watts, Catrina T. White, Thomas V. Rousselle, Ankush Gosain, Amandeep Bajwa, Joan C. Han, Kent A. Willis, Joseph F. Pierre

**Affiliations:** 1 Department of Pediatrics, College of Medicine, The University of Tennessee Health Science Center, Memphis, TN USA; 2 Department of Geography and the Environment, Villanova University, Radnor, PA USA; 3 Department of Ecological Science, Vrije Universiteit Amsterdam, Amsterdam, The Netherlands; 4 Department of Surgery, Transplant Research Institute, James D. Eason Transplant Institute, College of Medicine, The University of Tennessee Health Science Center, Memphis, TN USA; 5 Division of Pediatric Surgery, Department of Surgery, College of Medicine, The University of Tennessee Health Science Center, Memphis, TN USA; 6 Department of Physiology, College of Medicine, The University of Tennessee Health Science Center, Memphis, TN USA; 7 Division of Neonatology, Department of Pediatrics, College of Medicine, The University of Alabama at Birmingham, Birmingham, AL USA; 8 Department of Microbiology, Immunology and Biochemistry, College of Medicine, The University of Tennessee Health Science Center, Memphis, TN USA

**Keywords:** Metabolism, Fungal host response, Obesity

## Abstract

As an active interface between the host and their diet, the gut microbiota influences host metabolic adaptation; however, the contributions of fungi have been overlooked. Here, we investigate whether variations in gut mycobiome abundance and composition correlate with key features of host metabolism. We obtained animals from four commercial sources in parallel to test if differing starting mycobiomes can shape host adaptation in response to processed diets. We show that the gut mycobiome of healthy mice is shaped by the environment, including diet, and significantly correlates with metabolic outcomes. We demonstrate that exposure to processed diet leads to persistent differences in fungal communities that significantly associate with differential deposition of body mass in male mice compared to mice fed standardized diet. Fat deposition in the liver, transcriptional adaptation of metabolically active tissues and serum metabolic biomarker levels are linked with alterations in fungal community diversity and composition. Specifically, variation in fungi from the genera *Thermomyces* and *Saccharomyces* most strongly associate with metabolic disturbance and weight gain. These data suggest that host–microbe metabolic interactions may be influenced by variability in the mycobiome. This work highlights the potential significance of the gut mycobiome in health and has implications for human and experimental metabolic studies.

## Abstract (web summary)

Tahliyah S. Mims et al. investigate the influence of the gut mycobiome abundance and composition on host metabolism. Using mice from four different commercial suppliers they find that the gut mycobiome is shaped by diet, and that abundance and composition correlate with key metabolic features. In particular they find that *Thermomyces* and *Saccharomyces* species most strongly associate with weight gain.

## Introduction

The modern diet is dominated by processed sugar and carbohydrates that are linked to metabolic and immune-mediated diseases^1^. Disruption of the gut microbiome also influences the development of metabolic disease^2,3^, and dietary composition is a key driver of gut microbial community structure and function^1^. Gut microbes form an interface between the diet and the host, participate in digestion and energy extraction from otherwise indigestible fiber and oligosaccharides^4^, produce short-chain fatty acids and novel metabolites, and ultimately shape host endocrine and immune signaling. Given these findings, robust microbial communities appear crucial component to metabolic homeostasis as supported by data across diverse species, life stages, and disease states^2,5^.

While the gut microbiome is often equated to bacteria, microbial communities contain diverse populations of archaea, viruses, protists, and fungi^6^. Collectively termed the mycobiome, gut fungal communities of molds and yeasts are crucial to maintaining gut homeostasis and systemic immunity^7^. Mounting evidence suggests other domains of life, such as viruses, can also influence host metabolic tone^8^. However, data describing the role of the mycobiome in host metabolism remain scarce^9,10^. Recent studies in humans and mice indicate commensal fungi have the potential to influence host metabolism directly^7,11–14^ and via alterations to bacterial community composition^15,16^. The latter interactions between bacteria and fungi are likely to yield greater insight into the complex microbial gut ecology and health.

The role of the gut mycobiome in host metabolism remains unresolved. This question is further obscured by the difficulty in discriminating environmental and transiently ingested fungi from true colonizers, especially in free-living and genetically heterogeneous human populations. Thus, we designed a study using specific pathogen-free mice with the same genetic background so that we could control for age, sex, and previous founder exposures. We also sourced these mice from four different vendors to vary their initial gut mycobiomes. Utilizing this design, we tested our hypothesis that the gut mycobiome would associate with host metabolic response to a processed diet, which is representative of typical westernized diets.

First, we found the gut mycobiome of laboratory mice differs dramatically between animal vendors. Using a combinatory approach, we then identified fungal populations that shifted in response to processed diet and identified key fungal taxa that may be linked to host metabolic alterations. We show baseline correlations in the gut mycobiome strongly associate with changes in host adiposity and serum metabolic biomarkers in response to a highly processed low-fat diet. Our results support the role of the gut mycobiome in host metabolic adaptation and have important implications regarding the design of microbiome studies and the reproducibility of experimental studies of host metabolism.

## Results

To determine if the gut mycobiome altered host metabolism, we studied 72 mice obtained in one shipment each from four vendors [Charles River Laboratories (CR), Envigo (ENV), The Jackson Laboratory (JAX), and Taconic Biosciences (TAC)]. We characterized the fungal and bacterial communities of the jejunum, the most diverse fungal population in the mouse gut^10^. We quantified fungal communities using ITS2 rDNA gene sequencing, the most reliable culture-independent technique for detecting mammalian-associated fungi^17^. For fungi, we obtained 1,121,549 total ITS2 reads, with an average of 11,104 reads per sample. For bacteria, we obtained 1,502,041 total 16S reads, with an average of 14,582 reads per sample.

### Interkingdom composition of murine diets

A fundamental question of gut mycobiome research is the extent to which fungal organisms detected by next-generation sequencing represent organisms present in the diet or true commensal colonization by organisms replicating within the gut lumen. Therefore, we sequenced the food pellets that arrived with the mice from their respective vendors as well as the standardized chow and processed diets that the mice were fed in our experiments. In addition, we detected no consistent environmental or procedural contamination in our sequencing controls, where *Fusarium* was detected at low levels in some samples. On arrival, diets from the vendors showed similar fungal (Supplementary Fig. 1) and multi-kingdom microbial composition (Supplementary Fig. 2), distinct from the experimental diets and different from jejunal microbial communities of arriving animals, suggesting the ITS DNA detected in food was introduced during food manufacturing and not representative of viable organisms or major source of fungi identified within the gut.

### Gut fungal communities cluster by vendor

**Fig. 1:**
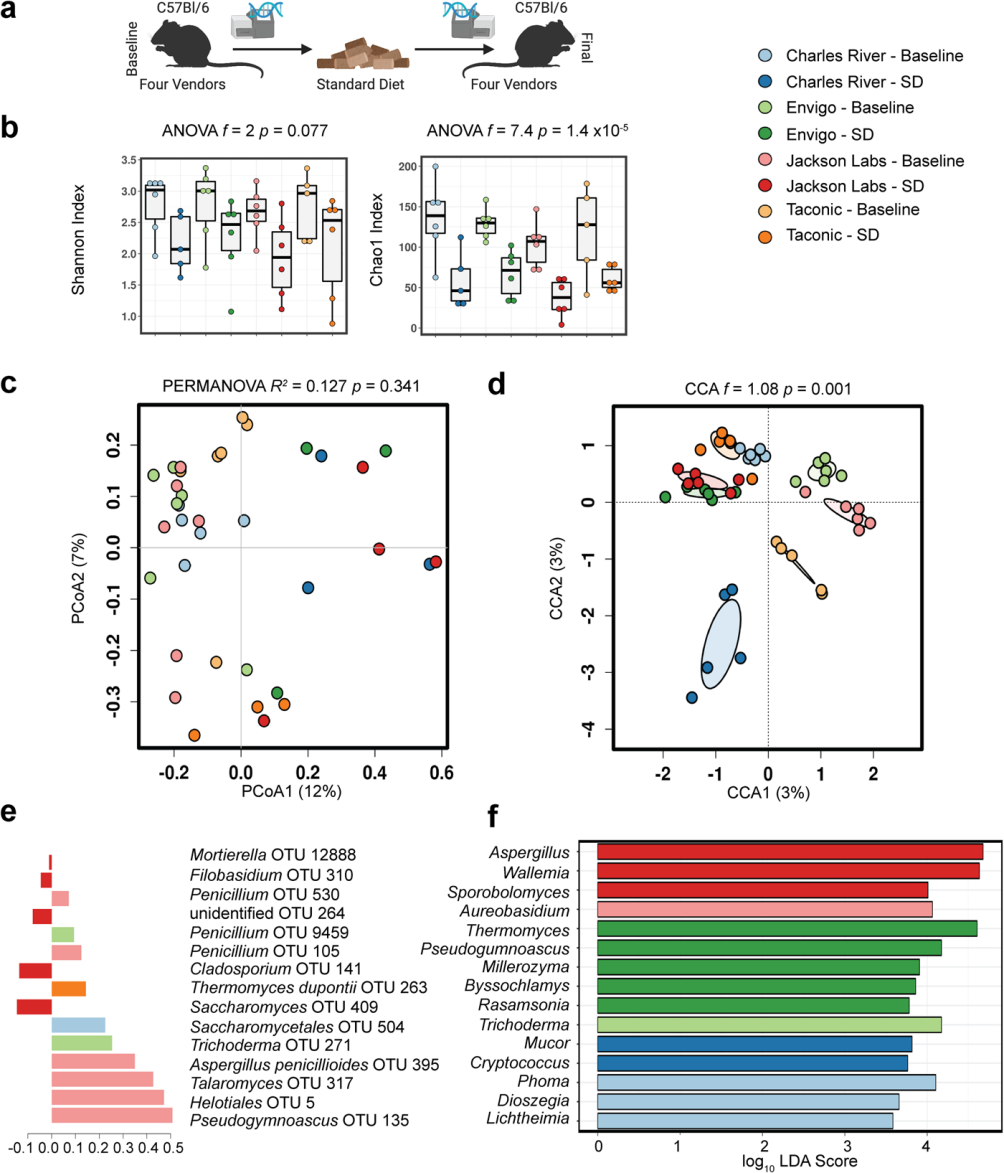
Gut fungal communities cluster by vendor, age, and dietary exposure. **a** Experimental schematic. **b** Compared to healthy mice exposed to a standardized chow diet for 8 weeks, baseline community diversity is higher in mice upon delivery from vendors. While gut fungal communities remained distinctly clustered by vendor, exposure to a standardized chow diet for 8 weeks exerted a convergent effect on community composition (**c**, **d** Bray–Curtis dissimilarity distance). Supervised partial least squares discriminant analysis (**e**) and linear discriminant analysis of effect size (**f**) confirm key operational taxonomic units and genera driving differences in community composition. Hypothesis testing was performed using ANOVA (**b**), PERMANOVA (**c**), and CCA (**d**). CCA canonical correspondence analysis, OTUs operational taxonomic units, SD standard diet, PERMANOVA permutational multivariate ANOVA, PCoA principal coordinates analysis. Schematic created using BioRender.com.

The gut bacterial communities of experimental mice differ markedly by vendor^18^. This variability has been utilized to understand the interrelatedness of gut and lung microbiota^19^ and standardize microbiota for genetic studies^20^. We tested to what extent the vendor-induced variability in gut bacterial communities held true for gut fungal communities. Upon arrival from the vendor, the gut fungal communities of healthy laboratory mice from each vendor were distinct (*p* = 0.001, *R*^2^ = 0.211, permutational multivariate analysis of variance, PERMANOVA; *p* = 0.575, permutational multivariate analysis of dispersion, PERMDISP2), with a unique composition (*p* = 0.001, *f* = 1.1; canonical correspondence analysis, CCA), despite similar diversity (*p* = 0.53, *f* = 0.77; ANOVA Chao1 Index, Fig. 1a–f). Across the four vendors, a core mycobiome of 18 genera was identified. An additional 14 genera were identified in animals from multiple vendors. Mice from JAX hosted the most unique genera (5: *Rasamsonia*, *Mycosphaerella*, *Millerozyma*, *Geosmithia,* and *Byssochlamys*) followed closely by TAC (*Bipolaris*, *Hanseniaspora,* and *Tritirachium*), then CR (*Lichtheimia* and *Dioszegia*), while only one genus (*Plectosphaerella*) was unique to animals from ENV (Supplementary Fig. 3). Interkingdom community composition was also analyzed, supporting large baseline community differences by vendor driven by unique bacterial (Supplementary Fig. 4).

### Gut fungal community diversity declines with age and exposure to standard diet

Data on longitudinal trends in gut mycobiome development are lacking. After we determined gut fungal communities in mice differed by their initial environment, we asked how these communities changed in a different environment. To determine the temporal dynamics of the gut mycobiome, we exposed mice with the same genetic background from different vendors to the same standardized chow diet and microenvironment for 8 weeks (Fig. 1a). While highly variable, the complexity of the gut microbial populations generally declines with maturity^21–23^, a finding we confirmed here in fungal populations. Community diversity declined in all animals exposed to standardized diet (*p* = 1.4 × 10^−5^, *f* = 7.4; Chao1 Index, Fig. 1b). Community composition similarly converged with time, resulting in more similar, less complex, but still distinct fungal communities (*p* = 0.341, *R*^2^ = 0.127; PERMANOVA. *p* = 0.215; PERMDISP2, Fig. 1c. *p* = 0.001, *f* = 1.08; CCA, Fig. 1d). Supervised partial least squares discriminant analysis confirmed key operational taxonomic units driving differences in community composition primarily result from loss of unique taxa (Fig. 1e, f). In general, fungal community differences between the vendors converged, resulting in communities with overall similar diversity and more similar community composition. A reduced core of 12 shared fungal genera remained, with animals from CR and Tac being the most divergent (Supplementary Fig. 5). As previously reported, bacterial communities vary by vendor^18^, consistent with our findings. In contrast to decreased fungal diversity, bacterial diversity increased after 8 weeks of standard diet, and beta diversity remained distinct (Supplementary Fig. 6 and Table 1). Bacterial changes were identified in the genus *S247, Adlercreutzia*, and *Desulfovibrio* in response to the standard chow diet. Given independent shifts in fungal and bacterial communities, we then investigated temporal dynamics of fungal and bacterial communities by building a series of generalized linear models using *mvabund* that revealed fungal and bacteria communities differ by diet and timepoint (Table 1 and Supplementary Fig. 7). Community diversity decreased with time, leading to 5 taxa being selected against and only one taxon, *Aspergillus*, persisting. In contrast, bacterial communities differed by vendor but not sex. From these analyses, we conclude jejunal fungal communities are indeed dynamic, selected by dietary intervention, and therefore susceptible to environmental influences. Collective interkingdom changes are reported in Supplementary Fig. 8, which were driven by shifts in the genus *S247, Clostridiales*, and *Desulfovibrio*.

**Table 1:** Determinants of microbial community composition in healthy mice.

Variable	Fungal		Bacterial	
	Likelihood ratio test^†^	*P*-value^‡^	Likelihood ratio test^†^	*P-*value^‡^
Vendor	237.9	0.153	874.5	0.032
Diet	132.5	0.004	843.5	0.001
Sex	159.5	0.036	288.8	0.756
Timepoint	252.3	0.001	333.3	0.007

^†^The likelihood ratio test quantifies the goodness-of-fit between generalized linear models (*mvabund*).

^‡^*P*-values were estimated using parametric bootstrapping with 999 permutations.

### Gut fungal community composition changes on exposure to processed diet

**Fig. 2:**
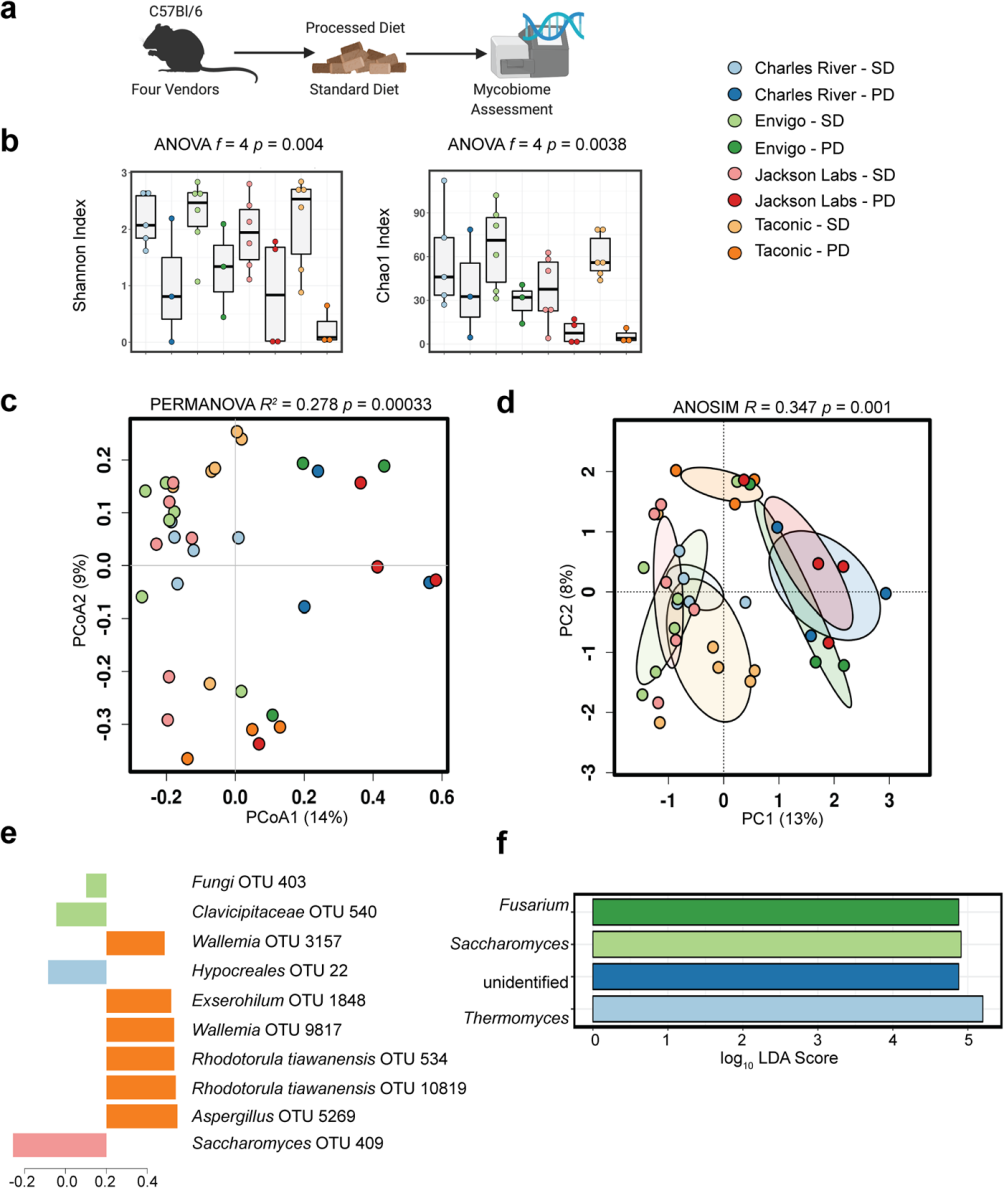
Exposure to processed diet produces a more pronounced alteration of gut fungal communities than does exposure to standardized chow. **a** Experimental schematic. **b** Compared to healthy mice exposed to a standardized chow diet for 8 weeks, mice exposed to a processed diet show reduced community diversity. While gut fungal communities remained distinctly clustered by vendor, exposure to processed diet for 8 weeks exerted a convergent effect on community composition that exceeded the similar effect of standardized chow (**c**, **d** Bray–Curtis dissimilarity distance). Supervised partial least squares discriminant analysis (**e**) and linear discriminant analysis of effect size (**f**) confirm key operational taxonomic units and genera driving differences in community composition. Hypothesis testing was performed using ANOVA (**b**), PERMANOVA (**c**), and ANOSIM (**d**). ANOSIM analysis of similarities, CCA canonical correspondence analysis, OTUs operational taxonomic units, LDA linear discriminant analysis, PERMANOVA permutational multivariate ANOVA, PD processed diet, PCA principal components analysis, PCoA principal coordinates analysis, SD standard diet. Schematic created using BioRender.com.

We demonstrated that environmental differences between gut fungal communities converged after exposure to a standardized diet and environment (Fig. 1). Given that gut bacterial communities are influenced by diet^24^, we sought to understand how the mycobiome was shaped by exposure to a processed diet. To address this question, we exposed the second group of mice from the same vendors to a processed diet for 8 weeks. Compared to baseline, the community diversity of fungal communities declined after exposure to processed diet (*p* = 0.0038, *f* = 4; ANOVA, Chao1 Index, Fig. 2a). The community composition was also markedly different (*p* = 0.00033, *R*^2^ = 0.278; PERMANOVA, Fig. 2b. *p* = 0.001, *R* = 0.347; analysis of similarities (ANOSIM), Fig. 2c, d, *p* = 0.004, *mvabund*, Table 1). Specific differences were detected by supervised partial least squares discriminant analysis (Fig. 2e) and linear discriminant analysis of effect size (Fig. 2f). This change resulted in a convergence of microbial communities that were no longer distinct by PERMANOVA and had similar overall diversity (Supplementary Fig. 9 and Table 1). Nonetheless, more sensitive generalized linear modeling identified significant differences before and after exposure to a processed diet (*p* = 0.001, *mvabund*, Table 1). We analyzed shifts in bacterial-specific community composition alone following 8 weeks of the processed diet. Similar to fungi, a processed diet significantly reduced the alpha diversity of bacterial communities (Supplementary Fig. 10). The processed diet also resulted in unique shifts across beta diversity explained by principal coordinate analysis axis 1 without community convergence observed in fungal populations and greater vendor-specific changes remained. Interkingdom community analysis supported these bacterial-specific differences, collectively suggesting the fungal community is more uniquely selected by the environment and dietary intake compared with bacteria (Supplementary Fig. 11).

### Gut fungal community composition differs between standard and processed diet

**Fig. 3:**
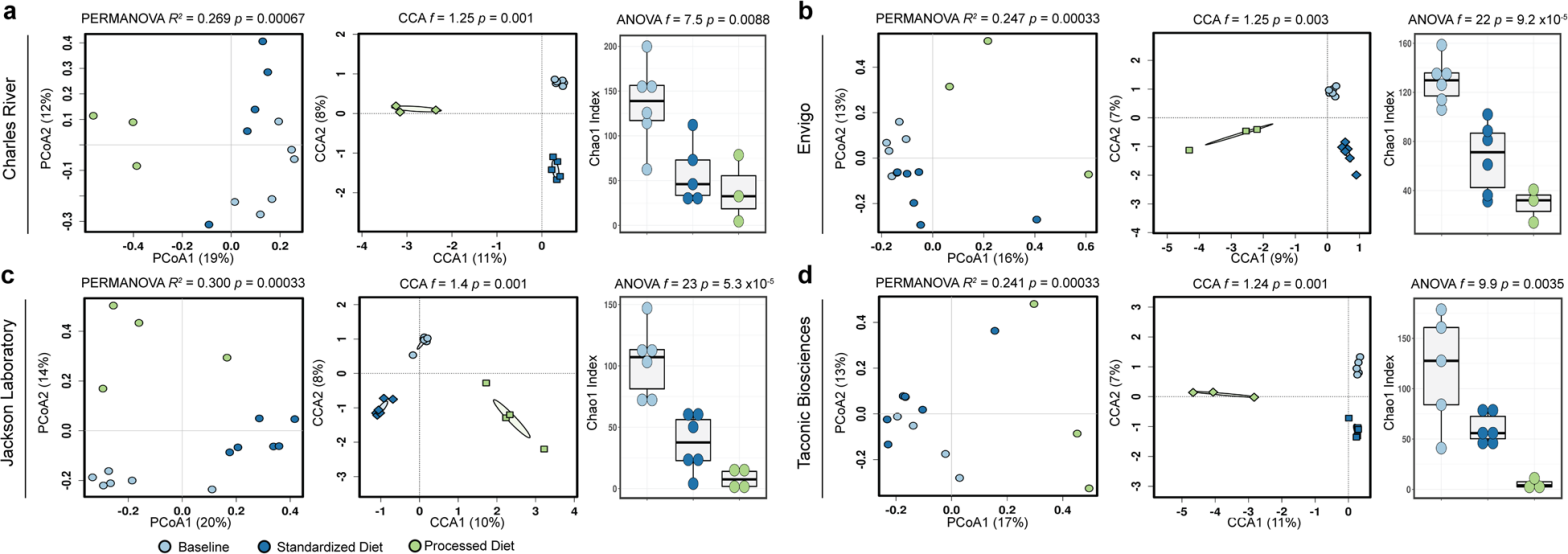
Baseline fungal composition influences final community composition. Differences in fungal community composition and diversity present on arrival from the four laboratory mouse vendors lead to persistent differences in the composition and diversity of fungal communities (**a**–**d**). Hypothesis testing for community composition was performed using permutational multivariate ANOVA and canonical correspondence analysis of Hellinger transformed Bray–Curtis dissimilarity distances and community diversity by ANOVA of the Chao1 index. CCA canonical correspondence analysis, OTUs operational taxonomic units, PERMANOVA permutational multivariate ANOVA, PCoA principal coordinates analysis.

We next asked how the fungal communities in mice exposed to processed diet differed from mice exposed to a standardized chow diet. While a similar overall trend in reduction in diversity and separation of multivariate clustering can be seen in all animals, differences between the community composition of each vendor remained (Fig. 3a–d). The diversity of animals from TAC and JAX was the most reduced, with CR having the most retained diversity. The clear divergence of processed diet exposed animals is also apparent on multivariate analyses (*p* = 0.01, *f* = 1.08; CCA, Fig. 3a–d). The shared core microbiome previously observed at baseline or after exposure to standardized chow collapsed with only one genus, *Aspergillus*, shared between all groups. Animals from CR had the most retained diversity, with the noteworthy unique prevalence of the genera *Candida* and *Aureobasidium* (Supplementary Fig. 9).

### Differences in fungal community composition persist despite exposure to processed diet

**Fig. 4:**
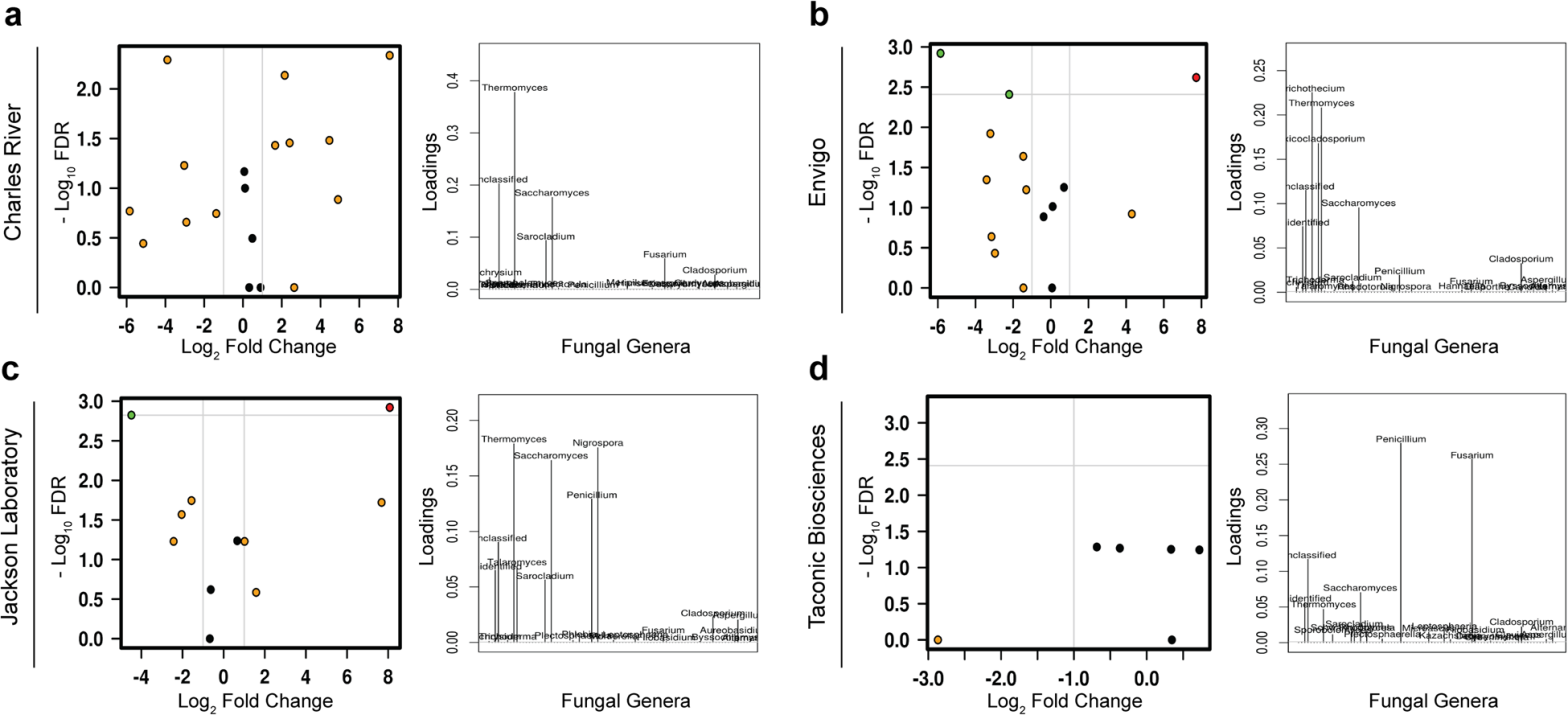
Differences among animals sourced from different vendors persist after exposure to processed diet. Volcano plots of mixed-effect regression identify differences between animals exposed to a processed diet that are highlighted by loading plots of discriminant analysis of principal components (**a**–**d**). FDR false discovery rate.

To identify differences between vendors after exposure to processed diet versus standardized chow, we utilized a series of complementary approaches, including analysis of the composition of microbiomes (ANCOM), mixed-effect regression and discriminant analysis of principal components. In animals from JAX, the most robust changes were a reduction in the genus *Saccharomyces* and corresponding bloom in *Thermomyces*, while animals from ENV demonstrated only the reduction in *Saccharomyces* and animals from CR the bloom in *Thermomyces*. Animals from TAC showed the opposite effect: *Thermomyces*, which was already at low levels in animals exposed to a standard diet, was eliminated after exposure to processed diet, as was the genus *Penicillium* (Fig. 4a–d). Furthermore, generalized linear modeling identified three specific taxa across taxonomic resolution that were associated with exposure to the processed diet: the orders Helotiales (*p* < 0.001) and Saccharomycetales (*p* = 0.049) and the genus *Wallemia* (*p* = 0.014, Supplementary Fig. 9).

### Exposure to processed diet reduces fungi-associated co-occurrence networks

**Fig. 5:**
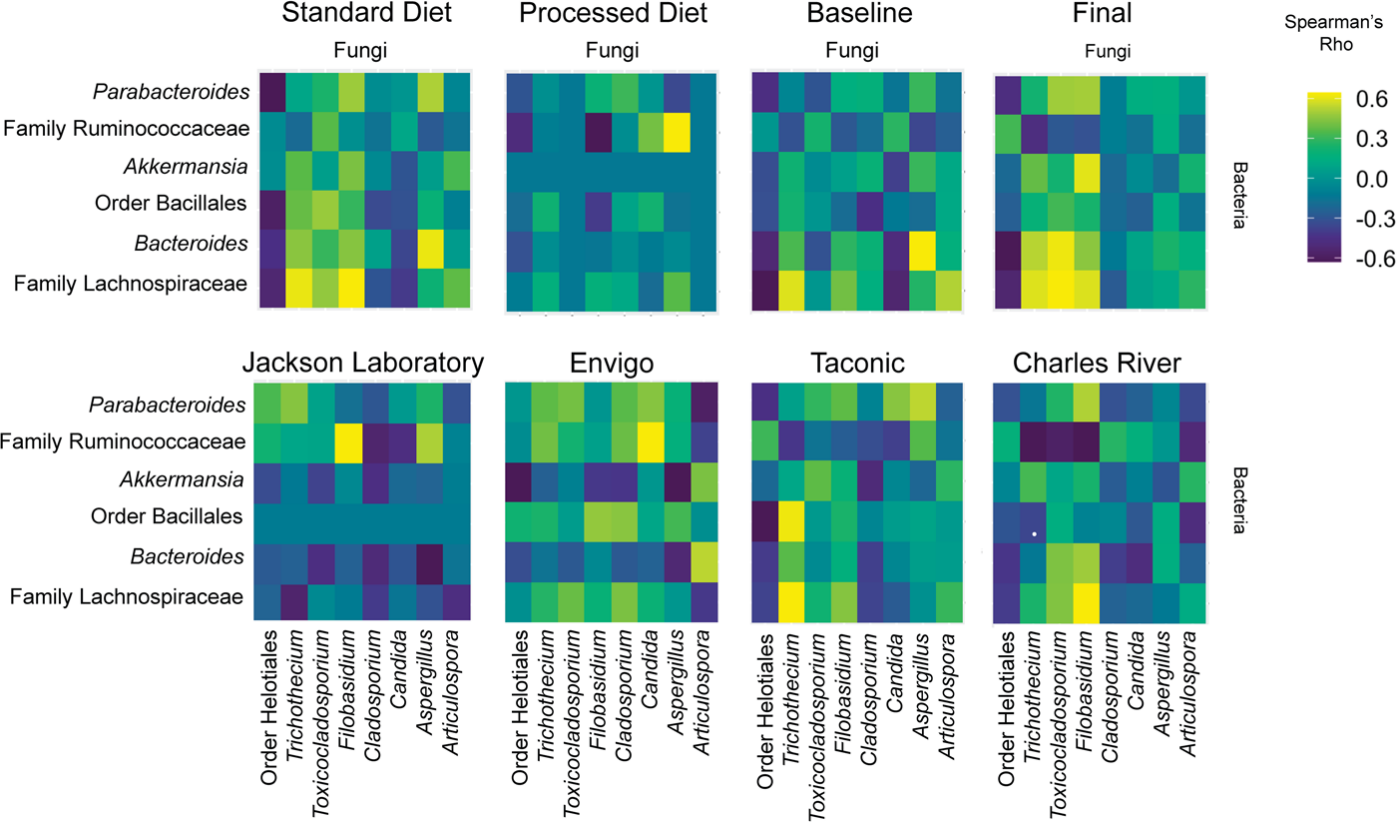
Interkingdom co-occurrence differs by vendor, diet, and time. The 14 most abundant taxa are displayed (comprising > 99.2% of variability). The color indicates the strength of Bonferroni corrected Spearman’s rho correlation coefficients. The *x* axis comprises fungal taxa with bacteria on the *y* axis. Complete co-occurrence networks are shown in Supplementary Fig. 11.

While overall interkingdom co-occurrence patterns among taxa did not significantly differ after exposure to the processed diet (*p* = 0.3438, *t*-test; Fig. 5), there was a noticeable reduction in the number and magnitude of co-occurrent relationships. This reduction was largely attributed to changes in four fungal taxa (Helotiales, *Trichothecium*, *Toxicocladosporium,* and *Filobasidium*).

### Gut fungal community composition correlates with differences in body composition and fat deposition

**Fig. 6:**
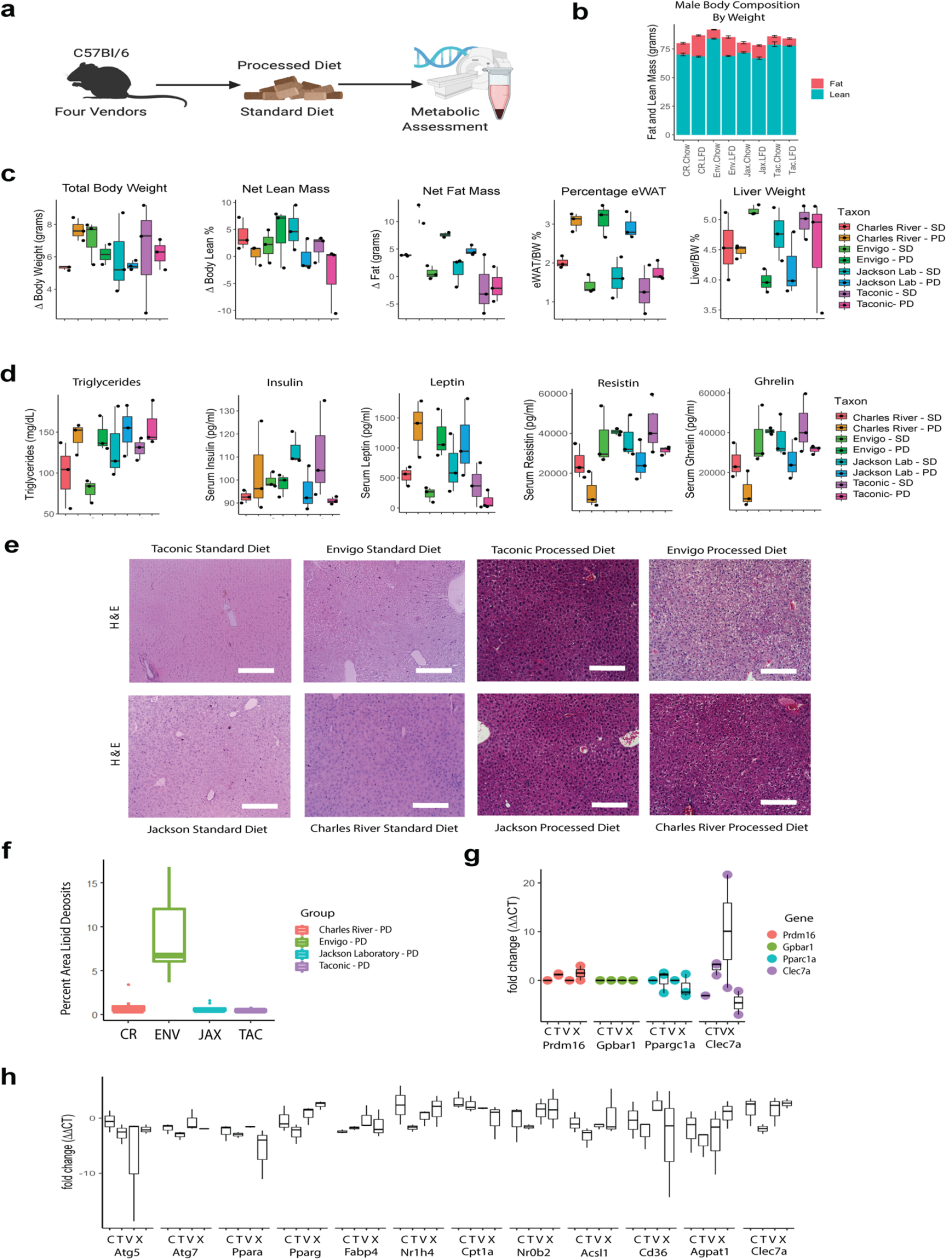
The metabolic phenotype of male mice is sensitive to gut fungal community composition. **a** Experimental schematic. **b** Male mouse body composition by EchoMRI. **c** Male mouse body composition by tissue collection. **d** Male serum metabolically active biomarkers. **e** Hematoxylin and eosin-stained liver tissue show increased deposition after a processed diet, particularly in mice from Envigo. **f** Quantification of lipid deposition in males exposed to processed diet. **g** Gene expression in eWAT under processed diet. **h** Gene expression in liver. eWAT epididymal white adipose tissue, PD processed diet, SD standard diet. Schematic created using BioRender.com.

Having established persistent differences in fungal community composition, we next asked if these changes altered host metabolic tone. Because female mice are protected from diet-induced obesity^25^, we focused on male mice (for results from female mice, see Supplementary Fig. 12). On the processed diet, with body composition quantified by EchoMRI and verified by tissue collection, male mice from CR, ENV and JAX gained fat mass, but mice from TAC did not (Fig. 6a–c). Utilizing an unbiased panel, we examined differences in serum metabolic biomarkers after a 5-h fast. Increases in fasting leptin and ghrelin and decreases in fasting resistin appropriately correlated with increased adiposity (Fig. 6d). While we were unable to detect lipid deposits in mice exposed to standardized diet, we noted complementary differences in hepatic steatosis (Fig. 6e, f), a marker of metabolic syndrome in mice^24^, especially in mice from ENV. Gut bacteria can influence host gene expression in animal studies^24^, so we also examined gene expression in metabolically active tissues to test if this was also true for fungi. In epidydimal white adipose tissue, differences between vendors were noted in *Prdm16* (PR/SET domain 16, Fig. 6g). In the liver, key differences were noted in *Ppara* (Peroxisome Proliferator Activated Receptor Alpha) family, *Nr1h4* (FXR, Farnesoid X Receptor), *Cd36* (CD36, cluster of differentiation receptor 36)*, Clec7a* (Dectin-1), and *Agapat1* (GPAT1, Glycerol-3-Phosphate Acyltransferase 1, Fig. 6h).

### Metabolic tone is strongly associated with variability in the gut mycobiome

**Fig. 7:**
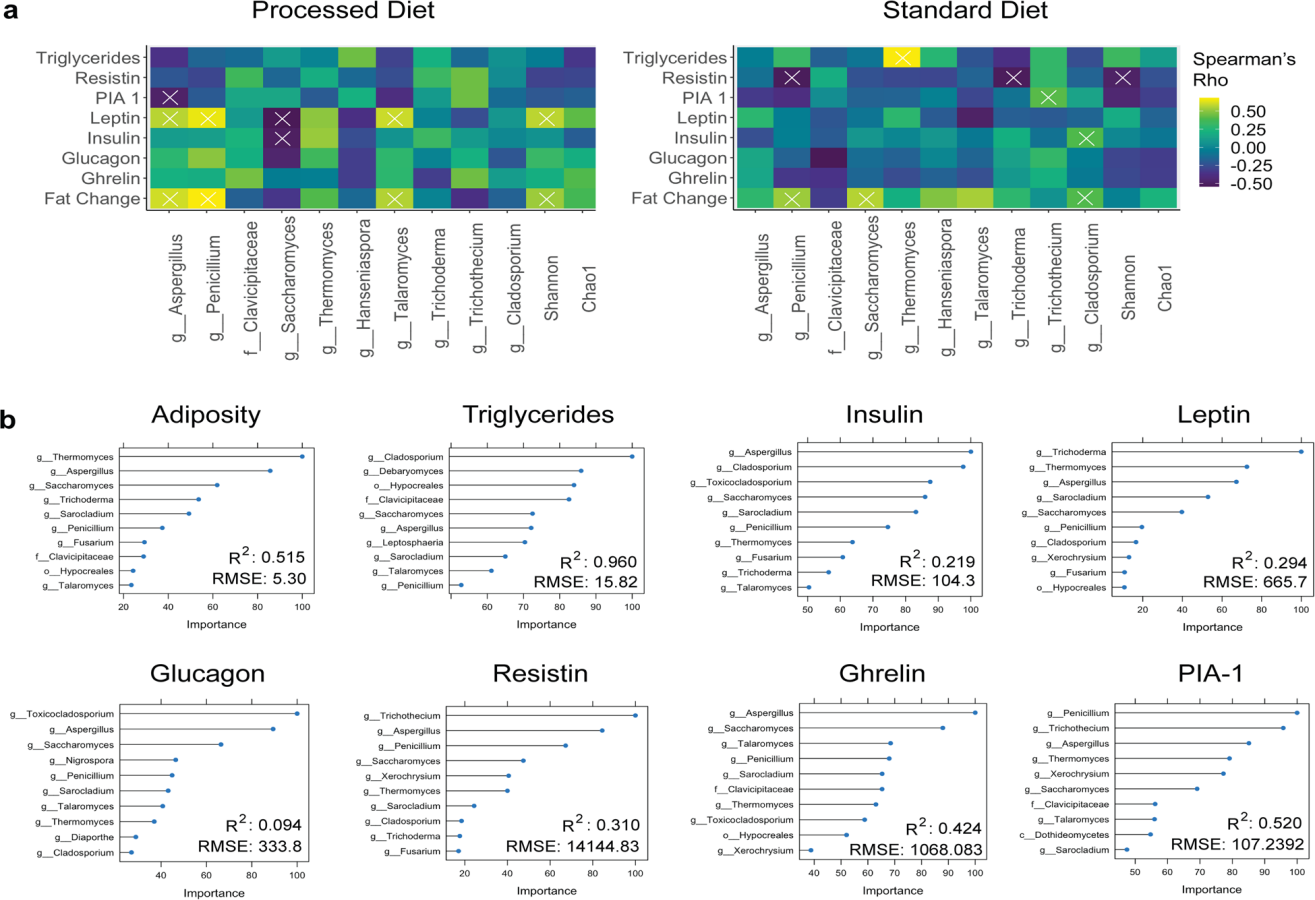
Fungal genera strongly associate with metabolic tone. **a** Biconjugate A-Orthogonal Residual method. X indicates *p* < 0.05 after false discovery rate correction. **b** Random forest regression models showing the relative importance of a particular taxon to the model. PIA-1 plasminogen activator inhibitor-1, *R*^2^ correlation coefficient, RMSE root mean square error.

To understand if the mycobiome was associated with differences in metabolic tone, we performed correlation analysis using the relative abundance of fungal genera and community diversity metrics with metabolic biomarkers (Biconjugate A-Orthogonal Residual, Spearman correlations with Bonferroni correction, Fig. 7a). Resistin and PIA-1 (plasminogen activator inhibitor-1) were negatively correlated, while and fat mass accrual was positively correlated with the presence of several key fungi. We further explored the relationship between fungal community composition and metabolic tone by building random forest machine learning models and performing variable importance analysis to identify key fungal taxa (Fig. 7b). We developed robust models for three metabolic outcomes that explain a sizable portion of the data variability. Differences in weight gain moderately correlated with differences in particular fungal taxa, of which the abundance and distribution of the genus *Thermomyces* were the most important. The most robust model was for serum triglyceride concentration, for which the genus *Cladosporium* was the most important taxa. Similarly, the genera *Saccharomyces* and *Aspergillus* were important for our model of fasting ghrelin concentration. Altogether, these results suggest fungal community composition is strongly associated with host metabolic tone.

## Discussion

Here, we investigated whether variations in gut mycobiome relative abundance and composition correlate with key features of host metabolism. This question drives towards understanding the complex interkingdom interactions between bacteria and fungi and how they are both collectively shaped and potentially contribute to host homeostasis. To address this question, we asked if differing starting mycobiomes could affect host adaptation to a standardized diet and an ultra-processed diet rich in purified carbohydrates in a manner that associated with deleterious metabolic outcomes. Our key findings are that the gut mycobiome of healthy mice is shaped by the environment, including diet, and significantly correlates with metabolic changes in the host. For instance, increased triglyceride concentrations and metabolic biomarkers, including the deposition of hepatic lipids, correlate with increased abundance of the fungal genera *Thermomyces* and decreased *Saccharomyces*. Our results highlight the potential importance of the gut mycobiome in health and have implications for human and experimental metabolic studies.

The bacterial microbiome is strongly influenced by dietary exposure^26^ and influences host metabolism^3^. Emerging work demonstrates dietary exposure also shapes fungal communities^9^. However, despite evidence for fungal pattern recognition receptors in the human gut^7^ and fungal influences on the disease in the human gut^27,28^, continuous gut colonization by fungi remains controversial in humans^29^. Convincing evidence indicates fungi colonize the mouse gut and influence host physiology^30^ and disease^31^. Diet may have a dominant effect over host genotype on the composition of the gut bacteriome with subsequent alterations in host-microbe interactions^32^. We observed a similar strong effect of diet on the composition of both fungal and interkingdom community composition. However, because we used mice with the same genetic background, we were able to examine the impact of the founding microbial communities largely independent of host genomic influences. In our study, both prolonged exposure to a processed diet and length of isolation in a specific pathogen-free environment reduced fungal diversity. In turn, reduced fungal diversity was associated with increased adiposity and physiologic alterations seen in the metabolic syndrome.

In our controlled study, we found key differences in baseline mycobiome composition reflect differences in metabolic tone in response to diet. For metabolic studies in mice, the choice of vendor, shipment, diet, and housing may have instrumental roles in shaping outcomes, which should encourage further caution in drawing causative relationships. Validating outcomes in mice from several vendors or across multiple shipments may be necessary to address this potential confounder. The implication for human microbiome studies, which often examine only bacteria and sample only fecal communities, is that the mycobiome may have unappreciated effects on microbiome-associated outcomes.

Exposure to a high-fat diet may alter fungal and interkingdom community composition^9^. Our work suggests complex alterations in co-abundance networks are associated with diet. Fungal cell wall components are a major point of interaction between fungi and bacteria in the environment^7^. For example, the abundance of a major gut bacteria, *Bacteroides thetaiotaomicron*, can be influenced by the presence of mannan in fungal cell walls^16^. Similarly, fungal chitin influences the composition of anaerobic bacteria^33^. Our co-occurrence correlation analysis suggests the loss of key fungi during dietary exposure was closely related to differences in bacterial community composition, which may represent niche replacement in the face of a shifting dietary environment or disruption of interkingdom metabolomic networks. This study extends the previous work^9^ in two important avenues. First, we examined the impact of a highly-processed diet on gut communities. This approach allowed us to identify more subtle physiologic changes in host metabolic tone. Second, by using mice from different vendors, we extended these observations by observing the effect of different founding mycobiomes on host metabolism.

Among the myriad metabolites and effectors that likely connect gut microbial communities to metabolism, the gut bacteriome may influence host metabolism through several major mechanisms^34^. While classically attributed to bacteria, fungi have an often underappreciated role in the production of metabolites and may interact with host physiology via analogous mechanisms.

One of the best-known fungal pattern recognition receptors in humans is Dectin-1 (*CLEC7A*), which recognizes fungal β-glucan. In addition to immune cells^7^, adipose tissue expresses Dectin-1^35^. In humans, obesity is associated with increased Dectin-1 expression in adipose tissue^35^. In mice, Dectin-1 (*Clec7a*) has a MyD88-independent role in diet-induced obesity^35^. In MyD88-deficient adipose tissue, Dectin-1 was upregulated in both adipocytes and adipose-associated macrophages. Furthermore, blockade of Dectin-1 led to improved glucose sensitivity and decreased numbers of CD11c^+^ macrophages. The reverse was also true in Dectin-1 activation^35^. We also observed differences in *Clec7a* expression in the liver that correlated with differences in adiposity. This may suggest that mycobiome-driven antigen-presenting cell education in the gut could directly influence systemic metabolic tone.

Another important function of gut microbes, often attributed solely to bacteria, is the production of secondary bile acids (BAs). Our understanding role of BAs has evolved from simple detergents to hormones intimately related to multiple metabolic processes, revealing important roles of BAs in dyslipidemia and type 2 diabetes^36^. BAs are a significant source of host–microbiome interaction via cellular receptors such as TGR5 (*Gpbar1*)^37^ and FXR (*Nrlh4*)^36^. Therefore, BA composition is interrelated with metabolic hormones such as leptin^38,39^, resistin^40^, ghrelin, GLP-1 (glucose-like peptide-1), and peptide YY^41^. However, fungi also produce BAs and likely participate in the gut BA pool to an underappreciated extent^42^. *Fusarium*, which we found was key to the structure of processed diet-induced weight gain-resistant TAC mice, is a prolific metabolizer of deoxycholic acid. Other key fungi, such as *Aspergillus* and *Penicillium*, can also produce secondary BAs^42^. BAs also influence the stability of other fungal metabolites. For example, luminal BAs^43^ influence the stability of a prominent lipase in *Thermomyces*, which we observed as the taxa most significantly associated with weight gain on a processed diet. Intriguingly, in our model, these differences correlated with differences in metabolic hormones, including leptin, resistin, and ghrelin. These findings may suggest a potential role for fungi in host metabolic processes via BA signaling.

In summary, these data indicate the gut mycobiome in healthy mice is highly variable and responds to disturbances such as changes in the environment and diet. Despite these ecological pressures, resilient differences in gut mycobiome composition in healthy mice strongly associated with differences in host metabolic tone, including differential fat deposition, metabolic biomarkers, and gene expression in metabolic tissues. We also highlighted two fungal-derived products with plausible effects on host metabolism. While there are potentially thousands of metabolically active fungal products, our work argues for future work in defined gnotobiotic animal models to further elucidate the mechanisms of mycobiome-host interaction. Finally, our findings suggest that differences in the gut mycobiome may be an underappreciated source of variability in health and metabolic outcomes in response to dietary interventions.

## Methods

### Ethics statement

All animal studies were approved by the Institutional Animal Care and Use Committee at the University of Tennessee Health Science Center (protocol # 17-089). Laboratory animal care policies follow the Public Health Service Policy on Humane Care and Use of Laboratory Animals. All results are reported in a manner consistent with ARRIVE guidelines^44^.

### Mice

This study was approved by the University of Tennessee Health Science Center Intuitional Animal Care and Use Committee. Eight-week-old specific pathogen-free C57Bl/6 mice of both sexes were purchased from Charles River Laboratories (CR, Wilmington, MA), Envigo (ENV, Indianapolis, IN), Taconic Biosciences (TAC, Rensselaer, NY), and Jackson Laboratories (JAX, Bar Harbor, ME). Mice were maintained in microisolators to prevent contamination. On arrival, 6 mice from each vendor were humanely euthanized, and jejunal contents were collected to quantify the baseline mycobiome. The remaining 12 mice/group were randomized to chow (Envigo 7912) or purified processed (Research Diets Inc. D12450B, New Brunswick, NJ) diet (Table 2) and monitored biweekly for 8 weeks to quantify food intake and body composition. Jejunal contents were collected to quantify the microbial communities at the end of the experiment. Details regarding exposure, housing, tissue collection, and sample processing are detailed in the online supplement. Animals were housed in sterile microisolators upon arrival in the same room under a standard 12-h light-dark cycle.

**Table 2:** Experimental diet composition.

Dietary components	Chow diet (Envigo 7912)	Processed diet (research diets D12450B)
Protein	19.1% kcal	20% kcal
Fat	5.8% kcal	10% kcal
Carbohydrate	44.3% kcal	70% kcal
Fiber	4.6%	–
Neutral detergent fiber	13.7%	–
Minerals	4%	4.74%
Energy density	3.1 kcal/g	2.82 kcal/g

### Fungal and bacterial ribosomal RNA extraction

Microbial DNA was extracted using lyticase and proteinase K, amplified, and sequenced according to our previously published protocols^45^. Sequencing was performed using the MiSeq platform (Illumina, San Diego, CA) for both the 16 S and the internal transcribed spacer region 2 (ITS2) ribosomal (r)DNA genes at the Argonne National Laboratory (Lemont, IL). Murine jejunal luminal samples were resuspended in 500 mL TNES buffer containing 200 units lyticase and 100 mL 0.1/0.5 (50/50 Vol.) zirconia beads. Incubation was performed for 20 min at 37 °C. Following mechanical disruption using ultra-high-speed bead beating, 20 mg proteinase K was added to all samples, which were incubated overnight at 55 °C with agitation. Total DNA was extracted using chloroform isoamyl alcohol, and total DNA concentration per mg stool was determined by qRT-PCR. Purified DNA samples were sent to the Argonne National Laboratory (Lemont, IL) for amplicon sequencing using the NextGen Illumina MiSeq platform, utilizing 16S rRNA MiSeq for bacteria and archaea and parallel ITS2 rDNA sequencing for fungi. Blank samples for the jejunal sequencing run passed through the entire collection, extraction, and amplification process remained free of DNA amplification.

### Body composition

Body composition (fat and lean mass) was measured biweekly using an EchoMRI 1100 system (EchoMRI, Houston, TX). System calibrations were performed before each session. These differences were confirmed by tissue collection after the experiment ended.

### Tissue collection

At the experimental endpoint, epididymal white adipose tissue depots (eWAT) and liver were immediately dissected and cleared of any connective tissue before being weighed to determine specific organ/tissue weights. Tissues were then snap-frozen in liquid nitrogen or fixed in 10% neutral buffered formalin for histology.

### Serum metabolic biomarkers

Serum triglycerides were quantified using a Triglyceride Colorimetric Assay Kit (Cayman Chemicals, Ann Arbor, MI). Metabolically active hormones were quantified using the Bio-Plex Pro Mouse Diabetes Panel (Bio-Rad Laboratories, Hercules, CA). Both were prepared according to the manufacturer’s instructions. Freshly obtained blood samples were allowed to clot for 30 min at room temperature before undergoing centrifugation at 5000 rcf for 5 min to obtain serum samples. We utilized an 8-plex Bio-Plex Pro Mouse Diabetes Panel from Bio-Rad (cat. #171F7001M, Hercules, CA) to quantify ghrelin, GIP, GLP-1, glucagon, insulin, leptin, PAI-1, and resistin in mouse serum.

### Histochemistry

Liver histology using hematoxylin and eosin was performed on paraldehyde-preserved tissue. The liver was harvested and fixed overnight in 4% paraformaldehyde. Tissues were processed (Tissue-Tek V.I.P, Sakura Finetek, Torrance, CA) and embedded in paraffin. For representative images, samples were cut (5.0 μm) and placed on adhesive coated slides (Newcomer Supply, Madison, WI), deparaffinized, rehydrated and H&E stained (Cat. No. H-3502, Vector Laboratories, Burlington, Canada). For pathology assessment of lipid deposition, samples were imaged in triplicate for representative images.

### Gene expression

Gene expression in liver and epididymal white adipose tissue was quantified using SYBR Green qRT-PCR (Applied Biosystems, Inc., Foster City, CA) on the QuantStudio 6 Flex Real-Time PCR System (Applied Biosystems). Tissues were collected in TRIzol reagent (Ambion, Austin, TX). RNA was isolated using the TRIzol and chloroform method, and RNA purity was validated through UV-Vis spectrophotometry using a Nanodrop Lite (ThermoScientific, Wilmington, DE). Total RNA (1.0 μg) was reverse-transcribed to complementary DNA (cDNA) using a Transcriptor First Strand cDNA Synthesis Kit (Roche, Indianapolis, IN) according to the manufacturer’s instructions. RT-PCR amplification comprised an initial denaturation step (95 °C for 10 min), 45 cycles of denaturation (95 °C for 10 s), annealing (55 °C for 20 s) and extension (60 °C for 30 s), followed by a final incubation at 55 °C for 30 s and cooling at 40 °C for 30 s. All measurements were normalized by the expression of the GAPDH gene, a stable housekeeping gene. Gene expression was determined using the delta–delta Ct method: 2−ΔΔCT (ΔΔCT = [Ct (target gene) − Ct(GAPDH)]tested − [Ct(target gene) − Ct(GAPDH)]control) and displayed as relative mRNA levels.

### Statistical analysis

Sequence data were processed in QIIME 1.9 and analyzed in Calypso 8.84^46^ (Hellinger transformed) and R 3.6.0 primarily using the *vegan* and *mvabund* (model-based analysis of multivariate abundance data) packages^47^. Biochemical data were analyzed using R. Machine learning was performed in R and presented in accordance with the TRIPOD guidelines^48^.

### Bioinformatics

Sequencing data were processed using QIIME 1.9.1. Sequences were demultiplexed, denoised, and clustered into operational taxonomic units (OTUs). For bacteria, sequences were aligned via PyNAST, and taxonomy was assigned against the SILVA database. For fungi, sequences were aligned, and taxonomy was assigned using the UNITE (dynamic setting) database. All OTU count data followed a negative binomial distribution. Processed data were then imported into Calypso 8.84 for further data analysis and visualization. Additional data analysis was performed in R. On import in Calypso, all mitochondrial sequences were discarded. For bacteria, any samples with <100 sequence reads were discarded, resulting in the removal of 0 samples from downstream analysis. For fungi, samples with <100 sequence reads were discarded, resulting in the removal of 21 presumed blank samples from downstream analysis. Processed data underwent a Hellinger transformation (square root of total sum normalization). We then utilized principal components analysis (PCA), principal coordinates analysis (PCoA), and canonical correspondence analysis (CCA) plots of Bray–Curtis dissimilarity distances to visualize beta diversity. Statistical significance of beta diversity clustering was then assessed using CCA and permutational multivariate analysis of variance (PERMANOVA) followed by a permutational analysis of multivariate dispersions (PERMDISP2) to assess the homogeneity of group variance (distance to centroid). To assess alpha diversity, we rarified bacterial samples to a depth of 4642 reads and rarefied fungal samples to a depth of 889 reads. Then, the Shannon and Chao1 diversity indices were calculated and differences assessed using ANOVA. On unrarefied data, we also performed univariate analyses using analysis of comparison of microbiomes (ANCOM), core microbiome analysis, supervised partial least squares discriminant analysis (sPLS-DA), mixed-effect regression with sex as a random effect, and negative-binomial regression (DESeq2 function) to quantify the differences in the relative abundance of specific microbial taxa, as appropriate. We used linear discriminant analysis of effect size (LEfSe) to perform high-dimensional biomarker identification. Co-occurrence networks using the top 15 most abundant taxa (representing 99.45% ± 1.3% of the interkingdom communities) were calculated using pairwise Spearman correlations with Bonferroni correction of taxon counts of which all were significant.

### Modeling

Generalized linear modeling at the multivariate and univariate (with Bonferroni correction) levels using a negative binomial distribution with likelihood ratio tests and resampled p-values to calculate significance was performed in R utilizing the mvabund package. Bacterial and Fungal OTUs were modeled separately to test the effects of vendor, diet, sex, and time of measurement of the microbiome and identify the taxa that significantly differ by variable.

Random forest regression (RFR) was used to regress changes in serology to variation in taxonomic interkingdom community composition. For this model, Hellinger transformed OTU counts were separated into 60% training and 40% validation sets with 10-fold cross-validations to test model accuracy. Data were preprocessed by removing zero and near-zero features, scaling and centering all features, and removing highly correlated features (rho > 0.90). Feature selection to identify the most important taxa in the random forest regression was conducted using the varimp (caret) function where a LOESS (locally estimated scatterplot smoothing) function is fit between the outcome and the predictor. We assigned an importance cutoff at 10 as the point when adding in new variables becomes asymptotic for all models (additional taxa do not increase model accuracy meaningfully). An R2 statistic was calculated for this model against the null model intercept. This number was returned as a relative measure of variable importance. Error was assessed by calculating the root mean standard error (RMSE), the average difference between the observed known values of the outcome and the predicted value by the model. This model was constructed and repeated for changes in fat, triglycerides, insulin, leptin, glucagon, resistin, ghrelin, and PIA-1 levels measured in the mice.

### Reporting summary

Further information on research design is available in the Nature Research Reporting Summary linked to this article.

## Supplementary information

Supplementary Information

Reporting Summary

## Data Availability

Sequences are available via the National Center for Biotechnology Information Short Read Archive (BioProject PRJNA597168).

## Code availability

Operational taxonomic unit, metadata tables, univariate analysis results, and R code are available from github.com/WillisLungLab/Vendors_gut_mycobiome.
