# Supplementary material for: The gut mycobiome of healthy mice is shaped by the environment and correlates with metabolic outcomes in response to diet
Source: Commun Biol. 2021 Mar 5;4:281. doi: 10.1038/s42003-021-01820-z (PMC7935979; doi:10.1038/s42003-021-01820-z)
Supplement: Supplementary file 1 — Supplementary Information [file 42003_2021_1820_MOESM1_ESM.pdf]

Supplemental Materials

The gut mycobiome of healthy mice is shaped by the environment and correlates with metabolic outcomes in response to diet

Tahliyah S. Mims, Qusai Al Abdallah, Justin D. Stewart, Sydney P. Watts, Catrina T. White, Thomas V. Rousselle, Ankush Gosain, Amandeep Bajwa, Joan C. Han, Kent A. Willis, Joseph F. Pierre

Supplemental Figure 1.

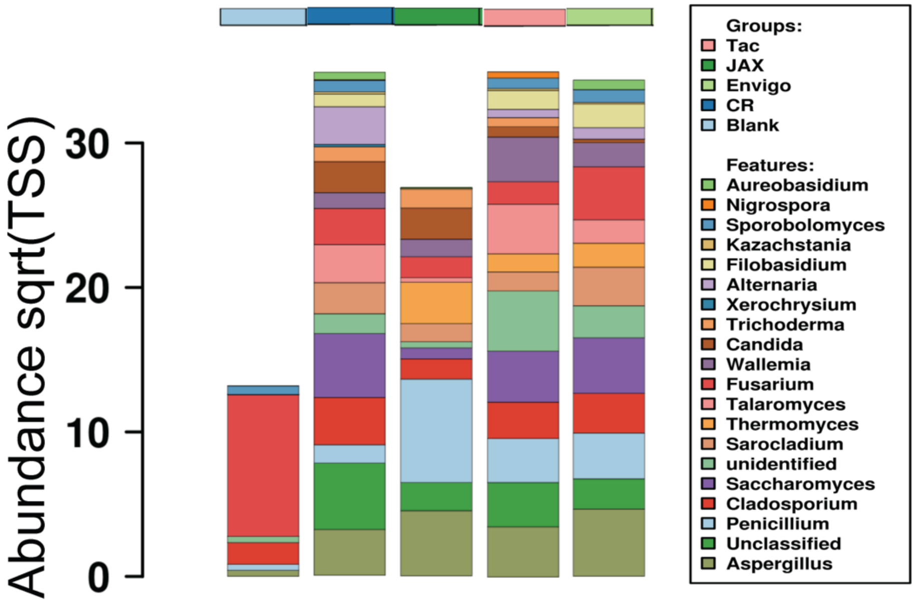

Supplemental Figure 1. Blank controls showed little amplification of ITS sequences, where *Fusarium* was present in low abundance. ITS sequencing of diet pellets provided during shipment showed similar fungal taxonomic composition, likely a result of food manufacturing processes.

Supplemental Figure 2.

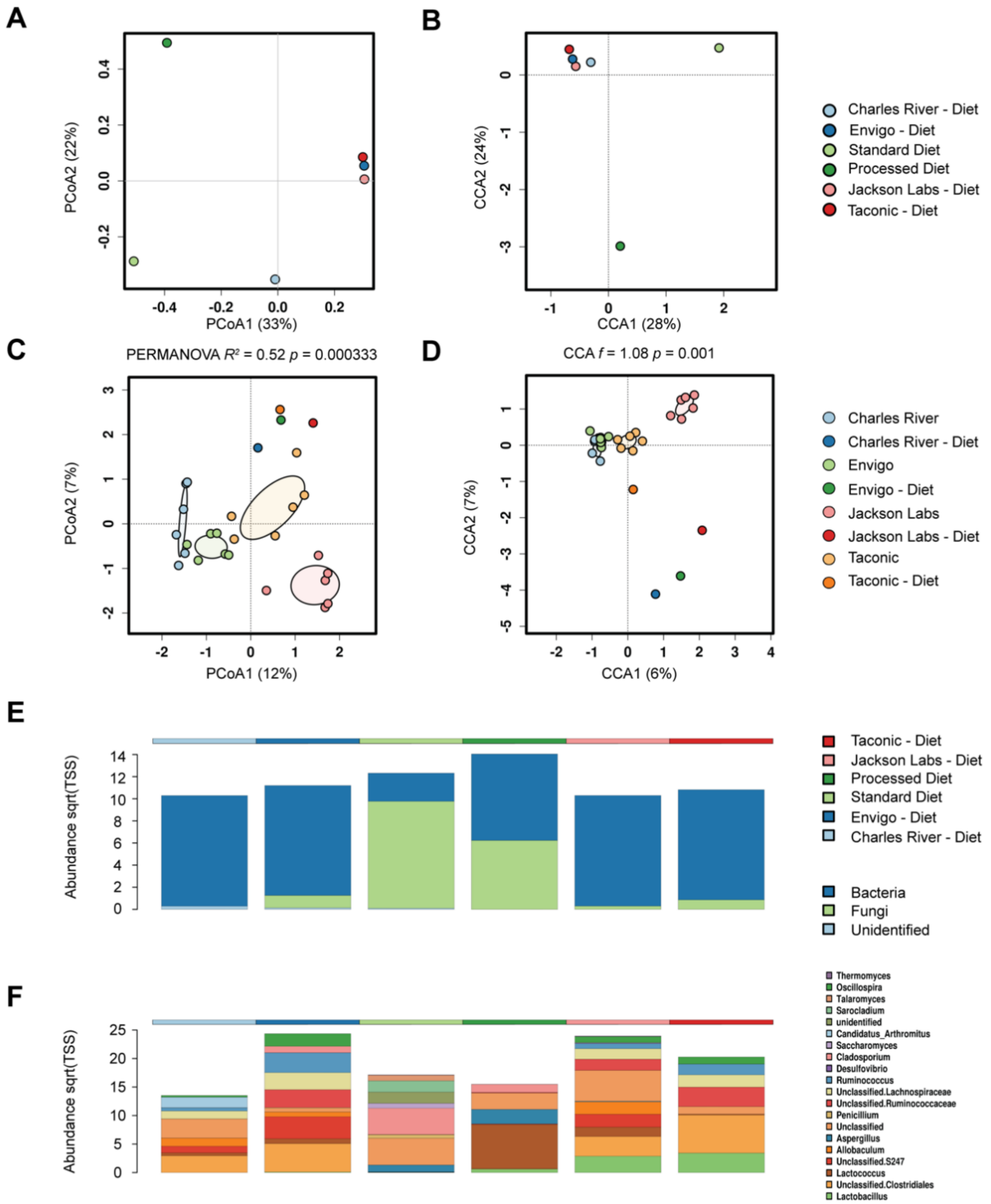

Supplemental Figure 2. Interkingdom (Bacteria + Fungi) composition of mouse diets obtained from vendors and in house diets. (a-b) Beta diversity was assessed in food pellets from vendors and diets used in house. (c-d) Beta diversity of vendor diet pellets and baseline jejunum samples from animals from each vendor. (e) Kingdom and (f) genus level taxonomy of diet pellets obtained from vendors and diets used in house. Hypothesis testing was performed using PERMANOVA (c), and CCA (d). CCA, canonical correspondence analysis; OTUs, operational taxonomic units; Diet, vendor provided chow; PERMANOVA, permutational multivariate ANOVA; PCoA, Principal coordinates analysis.

Supplemental Figure 3.

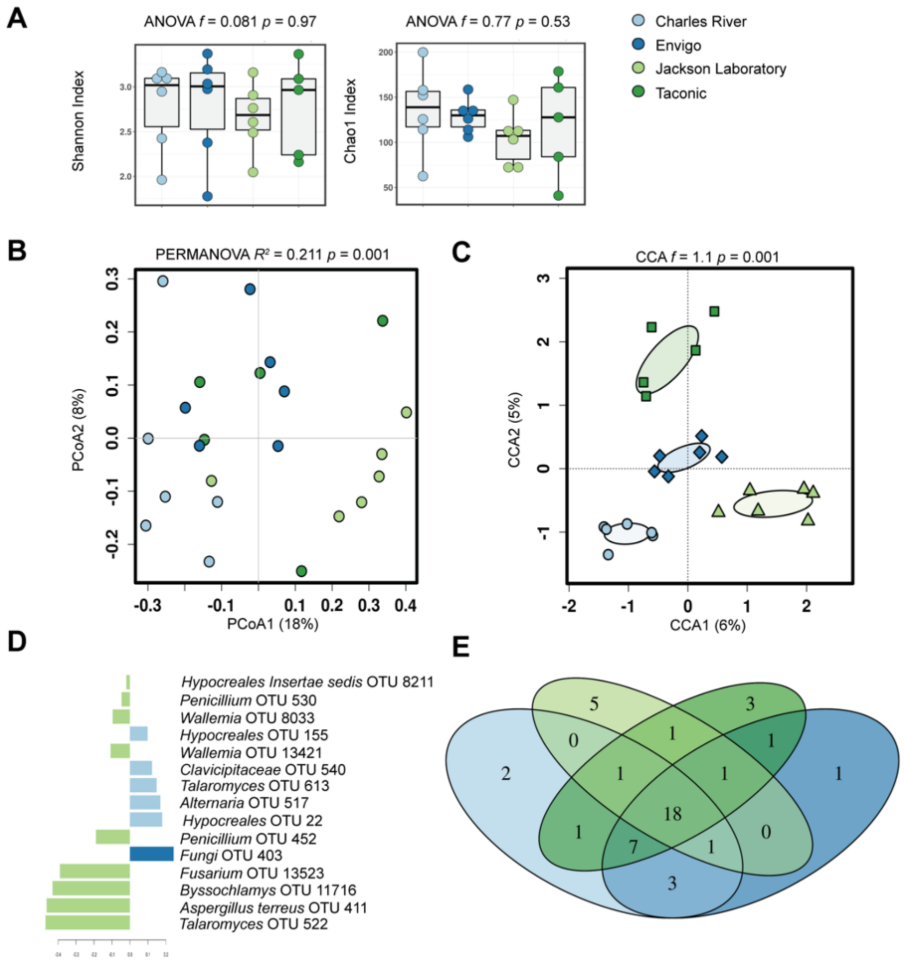

Supplemental Figure 3. The baseline fungal community composition differs between vendors. (a) Fungal community alpha diversity was similar between vendors. (b-c) Beta diversity showed distinct clustering of fungal communities between vendors. (d) Supervised partial least squares discriminate analysis for enriched fungal OTUs in each vendor. (e) Core microbiome of fungal taxa between vendors with 0.40 similar membership. Hypothesis testing was performed using ANOVA (a), PERMANOVA (b), and CCA (c). CCA, canonical correspondence analysis; OTUs, operational taxonomic units; PERMANOVA, permutational multivariate ANOVA; PCoA, Principal coordinates analysis.

Supplemental Figure 4.

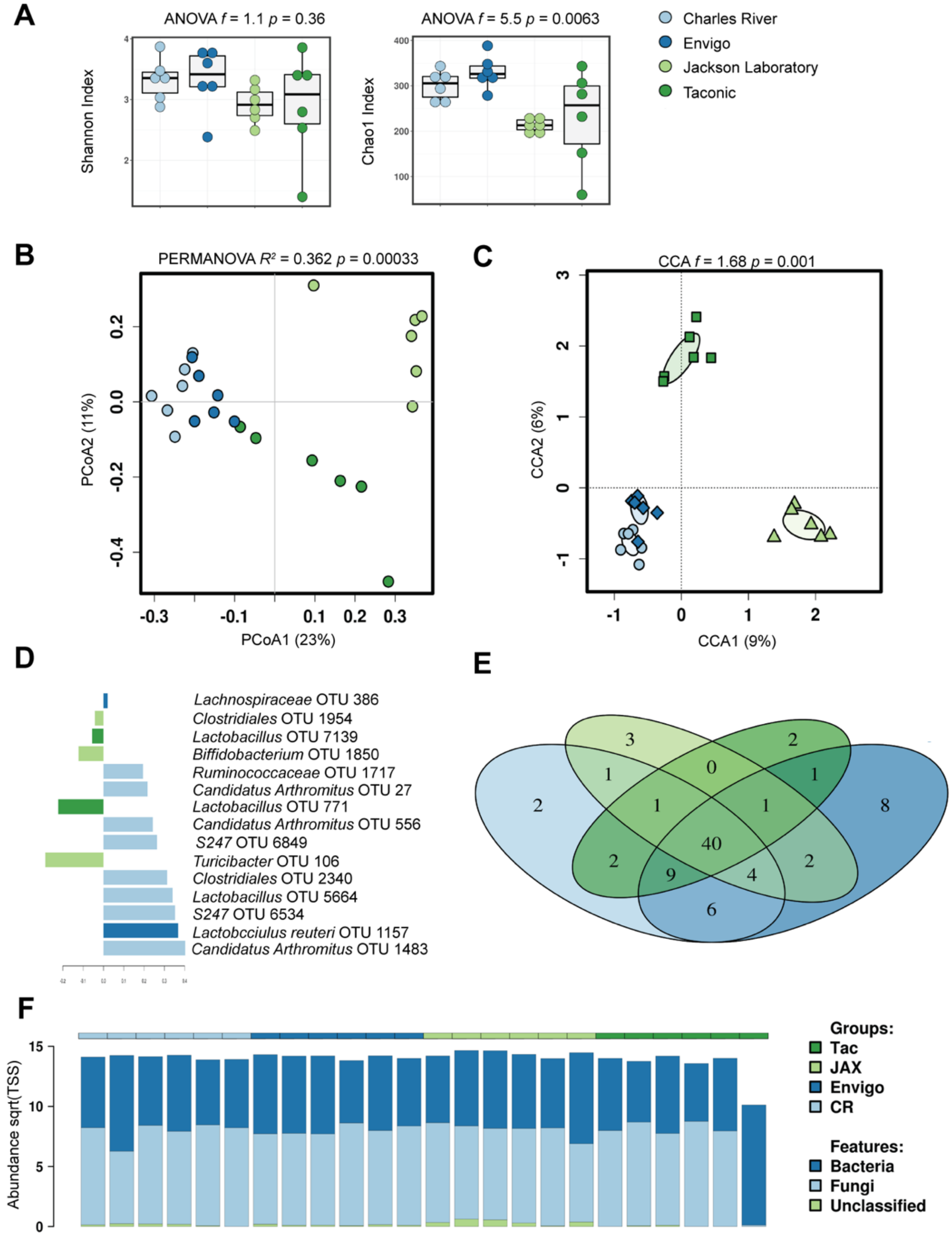

Supplemental Figure 4. The baseline interkingdom (Bacteria + Fungi) community composition differs between vendors. (a) Alpha diversity of interkingdom communities between vendors. (b-c) Beta diversity of interkingdom communities. (d) Supervised partial least squares discriminate analysis on interkingdom communities. (e) Core microbiome of interkingdom taxa between vendors with 0.40 similar membership. (f) Kingdom level abundance of OTUs across vendor samples at baseline. Hypothesis testing was performed using ANOVA (a), PERMANOVA (b), and CCA (c). CCA, canonical correspondence analysis; OTUs, operational taxonomic units; PERMANOVA, permutational multivariate ANOVA; PCoA, Principal coordinates analysis

Supplemental Figure 5.

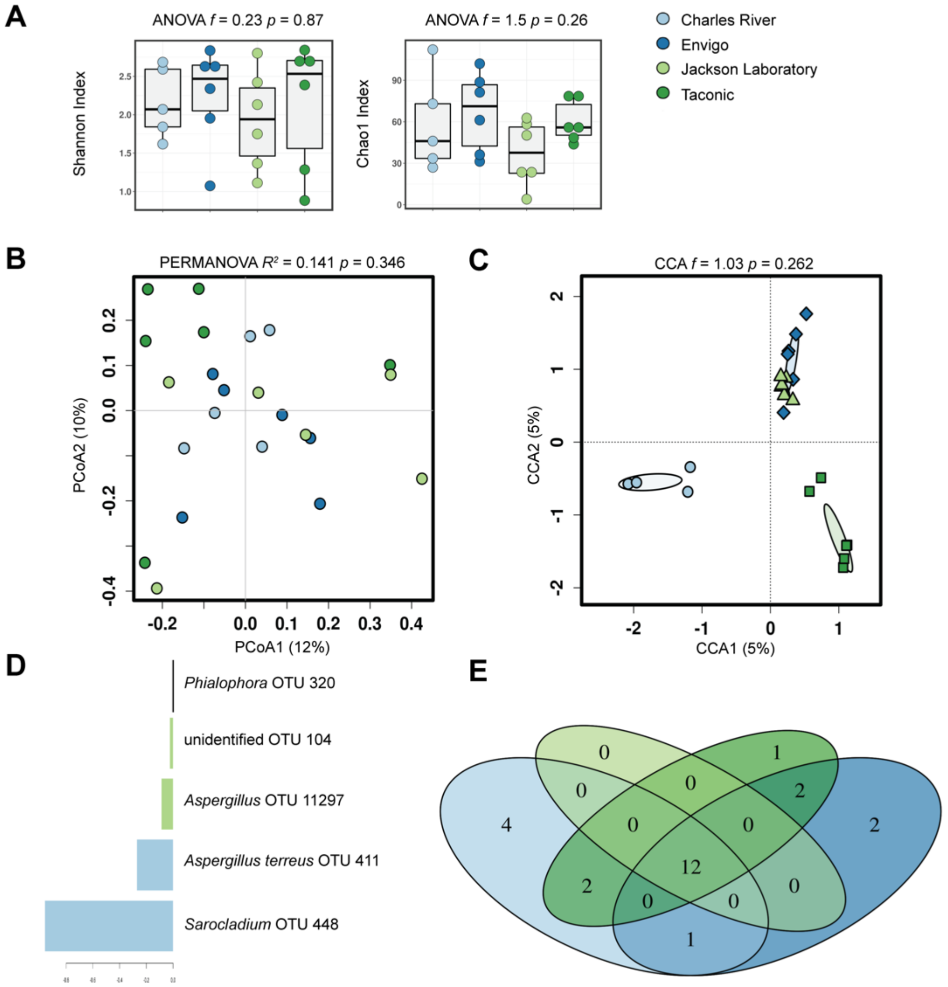

Supplemental Figure 5. Fungal community composition after 8-week exposure to 8 weeks of standardized diet. (a) Alpha diversity of fungal communities between vendors. (b-c) Beta diversity of fungal communities. (d) Supervised partial least squares discriminate analysis on fungal taxa. (e) Core microbiome of interkingdom taxa between vendors with 0.40 similar membership. Hypothesis testing was performed using ANOVA (a), PERMANOVA (b), and CCA (c). CCA, canonical correspondence analysis; OTUs, operational taxonomic units; PERMANOVA, permutational multivariate ANOVA; PCoA, Principal coordinates analysis.

Supplemental Figure 6.

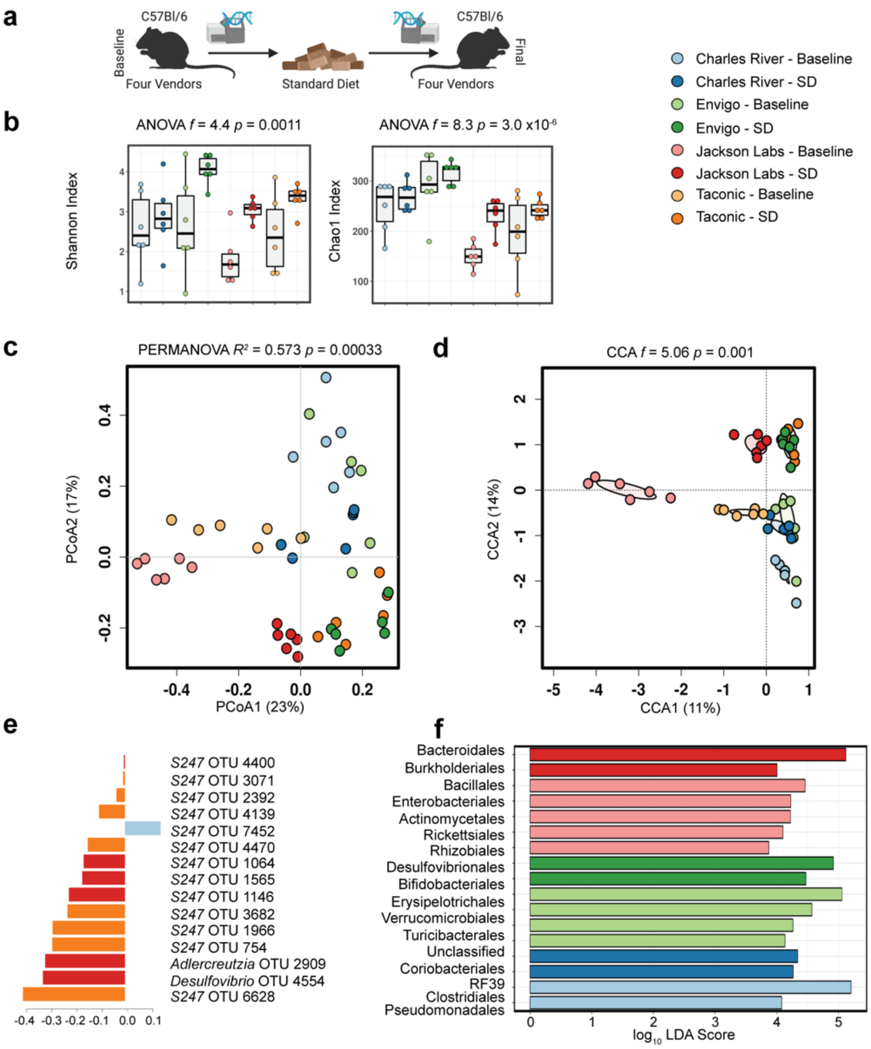

Supplemental Figure 6. Gut bacterial communities cluster by vendor, age and dietary exposure. (a) Experimental schematic. (b) Compared to healthy mice exposed to standardized chow diet for 8 weeks, baseline community diversity generally increased compared with mice upon delivery. Similar to fungi, bacterial communities remained distinctly clustered by vendor, exposure to standardized chow diet for 8 weeks exerted a convergent effect on community composition (c and d, Bray-Curtis dissimilarity distance). Supervised partial least squares discriminant analysis (e) and linear discriminant analysis of effect size (f) confirm key operational taxonomic units and genera driving differences in community composition. Hypothesis testing was performed using ANOVA (b), PERMANOVA (c) and CCA (d). CCA, canonical correspondence analysis; OTUs, operational taxonomic units; SD, standard diet; PERMANOVA, permutational multivariate ANOVA; PCoA, Principal coordinates analysis. Schematic illustrated using BioRender.

Supplemental Figure 7.

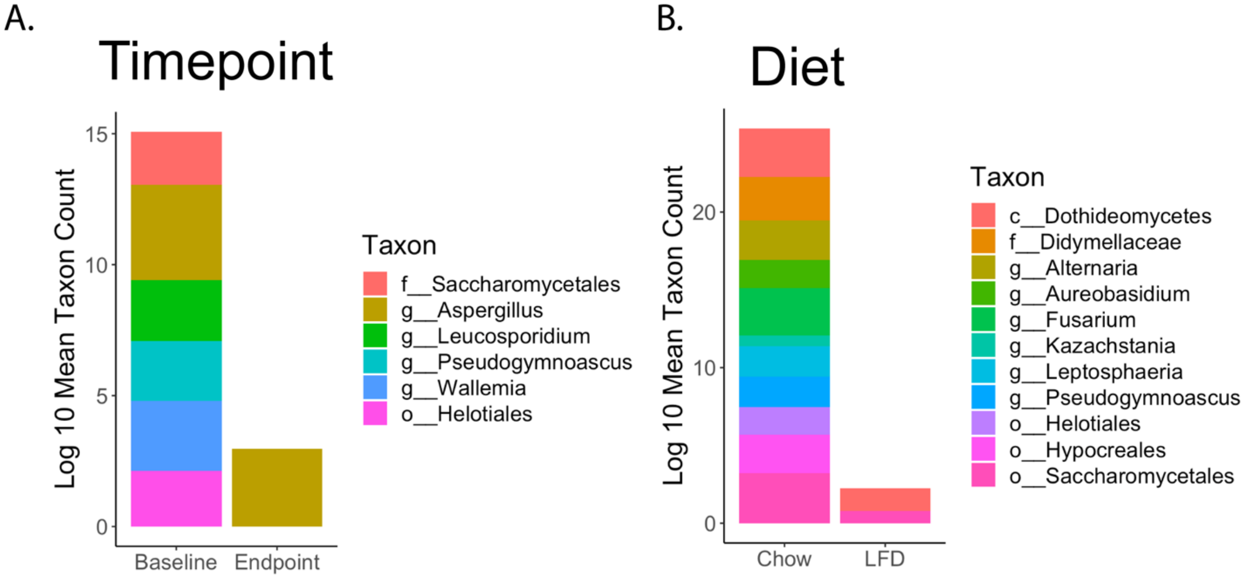

Supplemental Figure 7. Stacked bar plots of significant univariate MVABUND modeling of taxa responses. A. Log_{10} of mean taxon abundance separated by baseline and endpoint measurements. B. Log_{10} of mean taxon abundance separated by diet.

Supplemental Figure 8.

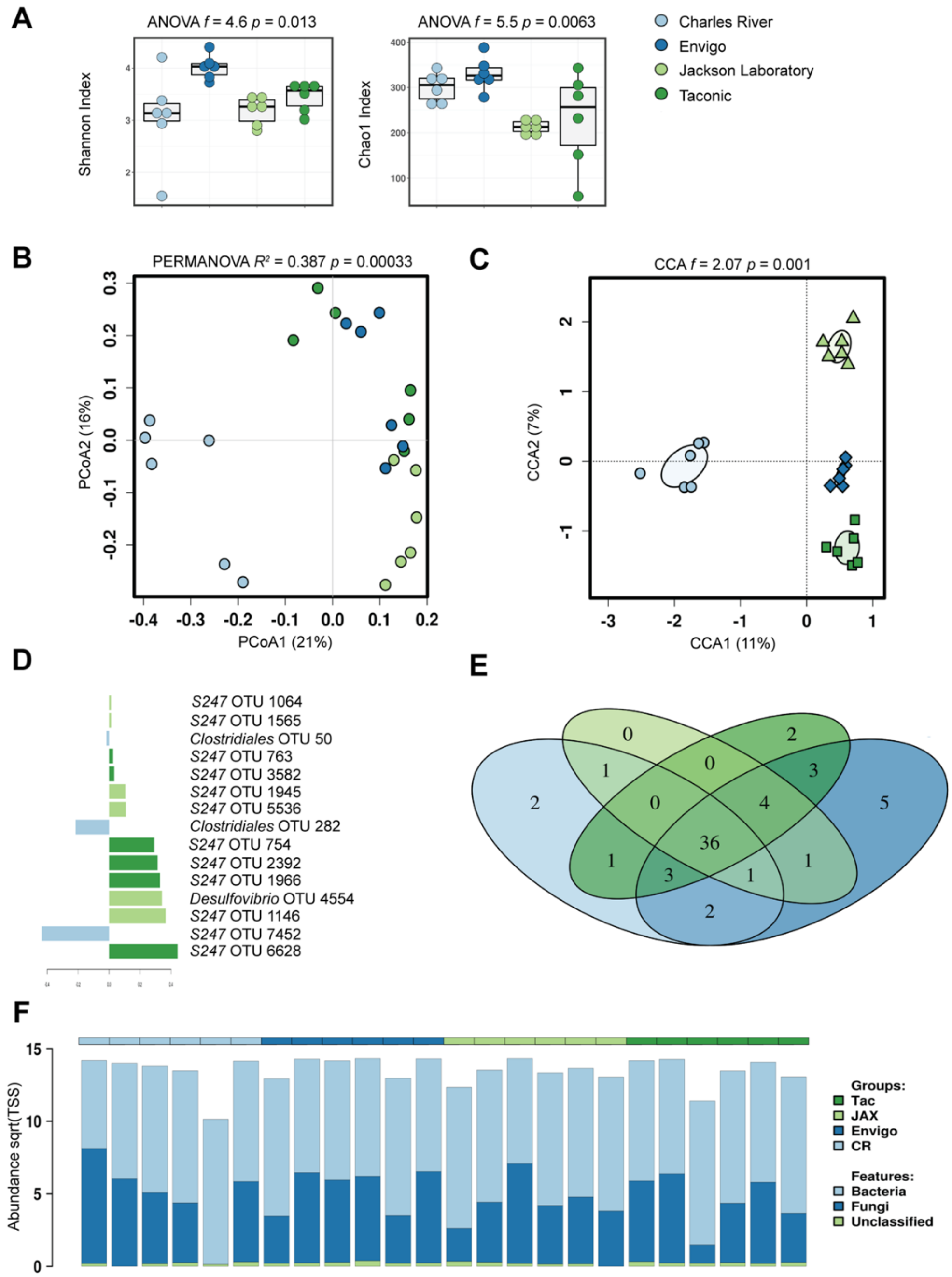

Supplemental Figure 8. Interkingdom (Bacteria + Fungi) community composition differs after exposure to 8 weeks of standardized diet. (a) Alpha diversity of interkingdom communities between vendors following 8 weeks of standard diet. (b-c) Beta diversity of interkingdom communities. (d) Supervised partial least squares discriminate analysis on interkingdom communities. (e) Core microbiome of interkingdom taxa between vendors with 0.40 similar membership. (f) Kingdom level abundance of OTUs across vendor samples at baseline. Hypothesis testing was performed using ANOVA (a), PERMANOVA (b), and CCA (c). CCA, canonical correspondence analysis; OTUs, operational taxonomic units; PERMANOVA, permutational multivariate ANOVA; PCoA, Principal coordinates analysis.

Supplemental Figure 9.

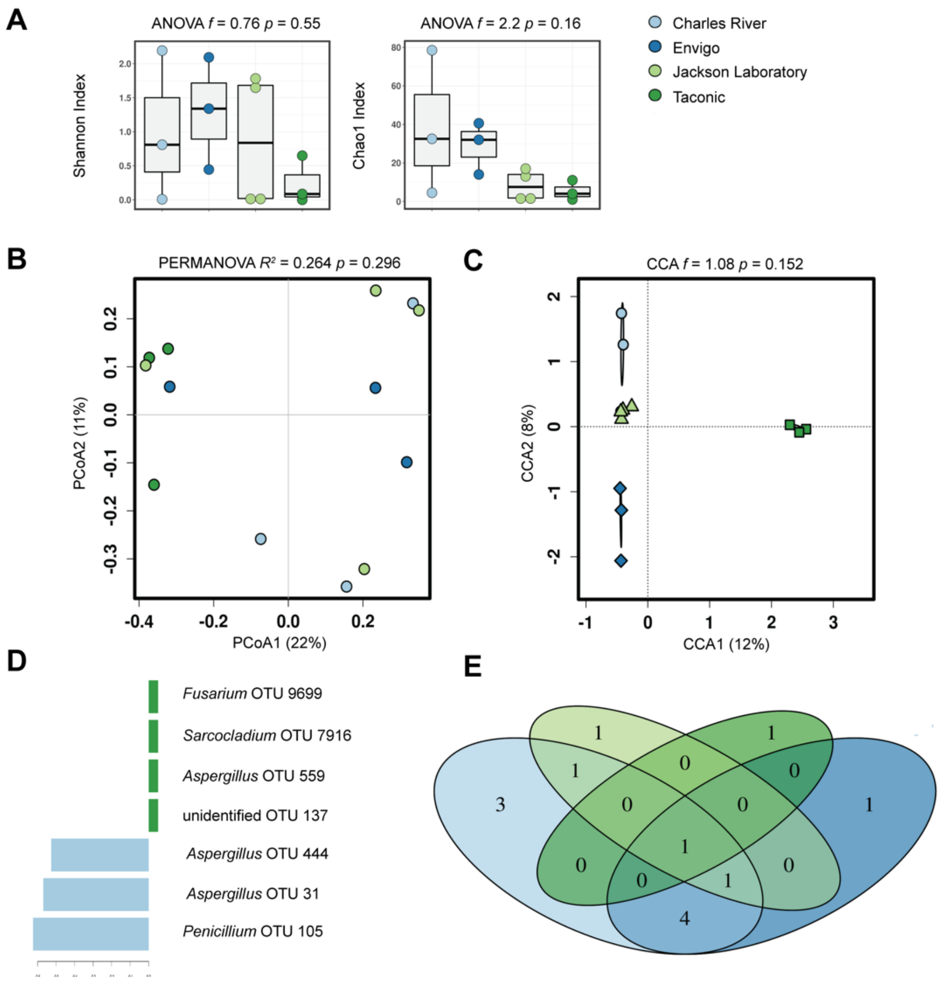

Supplemental Figure 9. Fungal community composition after 8-week exposure to processed diet. (a) Alpha diversity of fungal communities between vendors. (b-c) Beta diversity of fungal communities. (d) Supervised partial least squares discriminate analysis on fungal taxa. (e) Core microbiome of interkingdom taxa between vendors with 0.40 similar membership. Hypothesis testing was performed using ANOVA (a), PERMANOVA (b), and CCA (c). CCA, canonical correspondence analysis; OTUs, operational taxonomic units; PERMANOVA, permutational multivariate ANOVA; PCoA, Principal coordinates analysis.

Supplemental Figure 10.

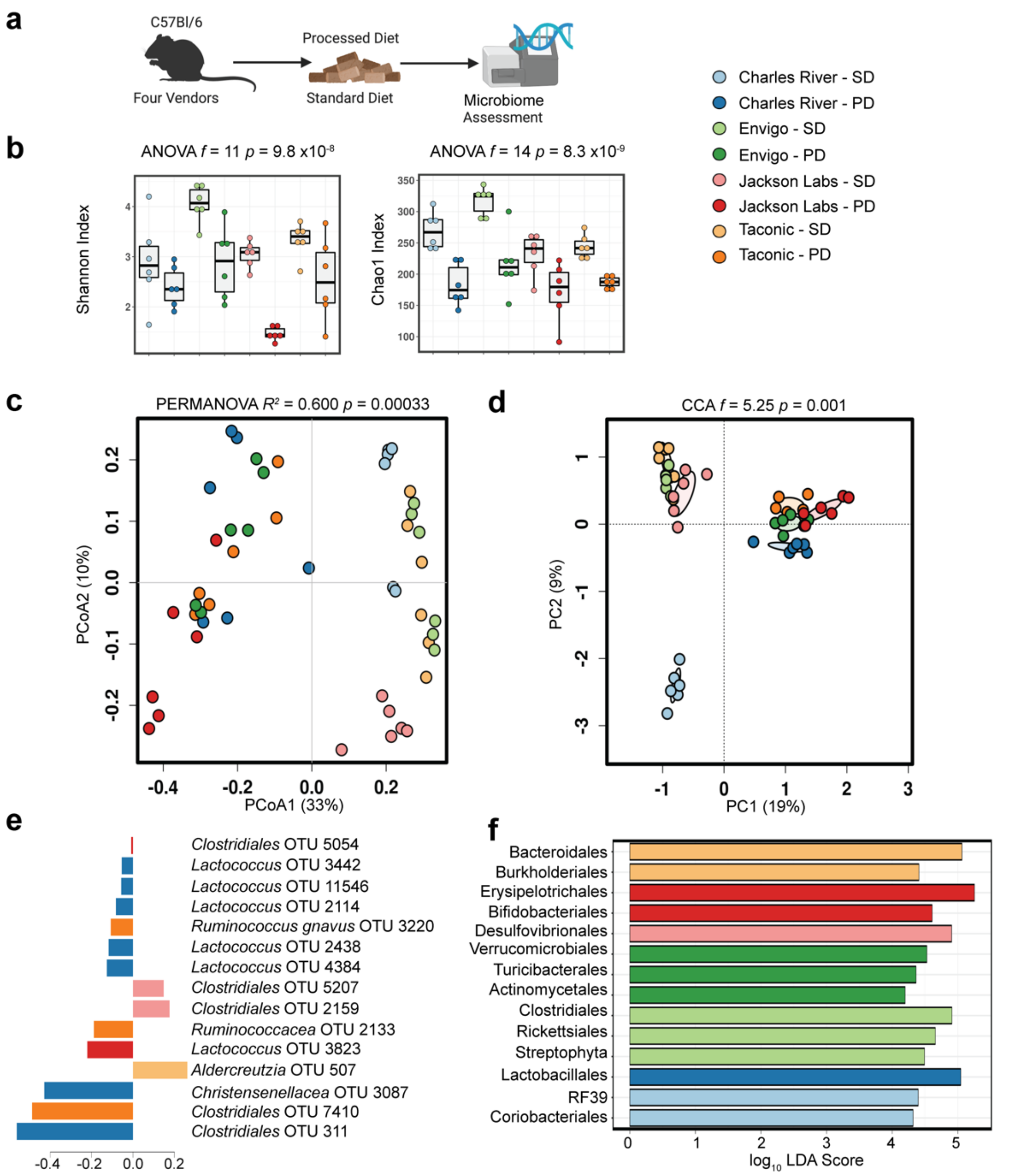

Supplemental Figure 10. Exposure to processed diet results in alteration of gut bacteria compared with standardized chow. (a) Experimental Schematic. (b) Compared to healthy mice exposed to standardized chow diet for 8 weeks, mice exposed to processed diet show reduced community diversity. While gut bacterial communities remained distinctly clustered by vendor, exposure to processed diet for 8 weeks exerted a convergent effect on community composition that exceeded the similar effect of standardized chow (c and d, Bray-Curtis dissimilarity distance). Supervised partial least squares discriminant analysis (e) and linear discriminant analysis of effect size (f) confirm key operational taxonomic units and genera driving differences in community composition. Hypothesis testing was performed using ANOVA (b), PERMANOVA (c) and ANOSIM (d). ANOSIM, analysis of similarities; CCA, canonical correspondence analysis; OTUs, operational taxonomic units; LDA, linear discriminant analysis; PERMANOVA, permutational multivariate ANOVA; PD, processed diet; PCA, principal components analysis; PCoA, principal coordinates analysis; SD, standard diet. Schematic illustrated using BioRender.

Supplemental Figure 11.

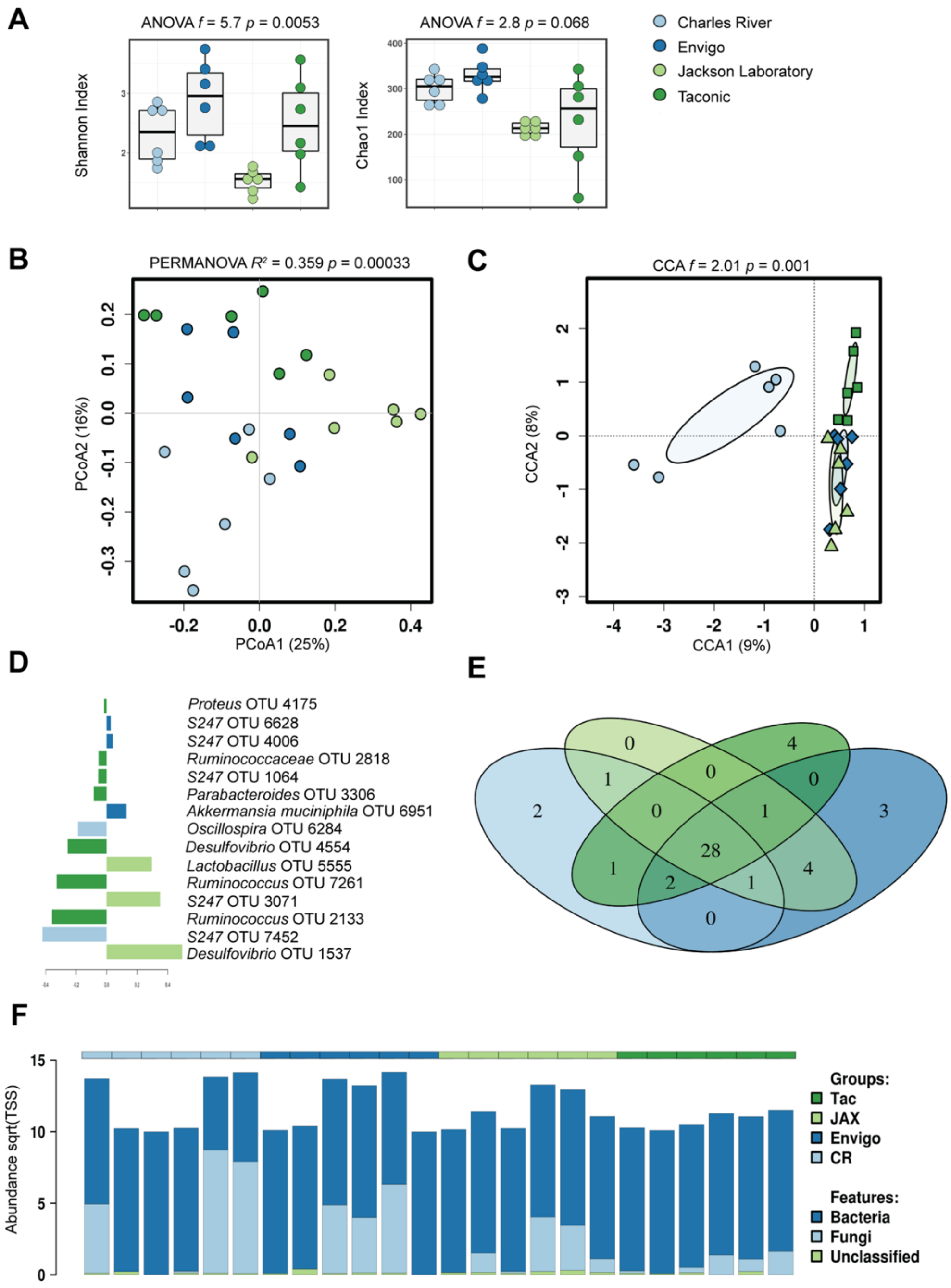

Supplemental Figure 11. The baseline interkingdom community composition differs after exposure to processed diet. (a) Alpha diversity of interkingdom communities between vendors following 8 weeks of processed. (b-c) Beta diversity of interkingdom communities. (d) Supervised partial least squares discriminate analysis on interkingdom communities. (e) Core microbiome of interkingdom taxa between vendors with 0.40 similar membership. (f) Kingdom level abundance of OTUs across vendor samples at baseline. Hypothesis testing was performed using ANOVA (a), PERMANOVA (b), and CCA (c). CCA, canonical correspondence analysis; OTUs, operational taxonomic units; PERMANOVA, permutational multivariate ANOVA; PCoA, Principal coordinates analysis.

Supplemental Figure 12.

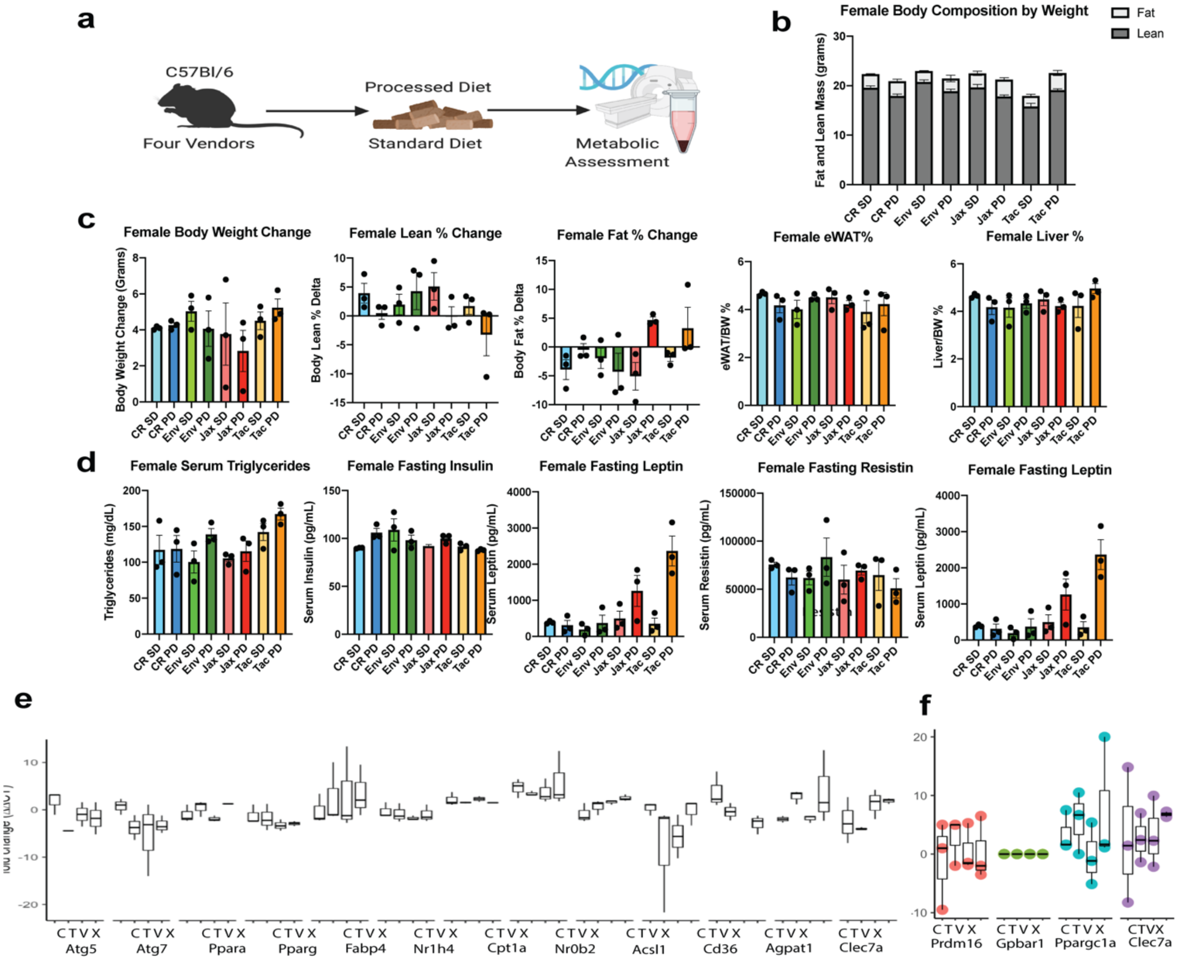

Supplemental Figure 12. Metabolic phenotype of female mice. (a) Experimental Schematic. (b) Female mouse body composition by EchoMRI. (c) Body weight change, Fat %, Lean %, normalized eWAT, normalized liver weight, (d) fasting serum triglycerides, insulin, leptin, resistin, ghrelin. (e) liver gene expression. (f) eWAT gene expression.

Supplemental Figure 13.

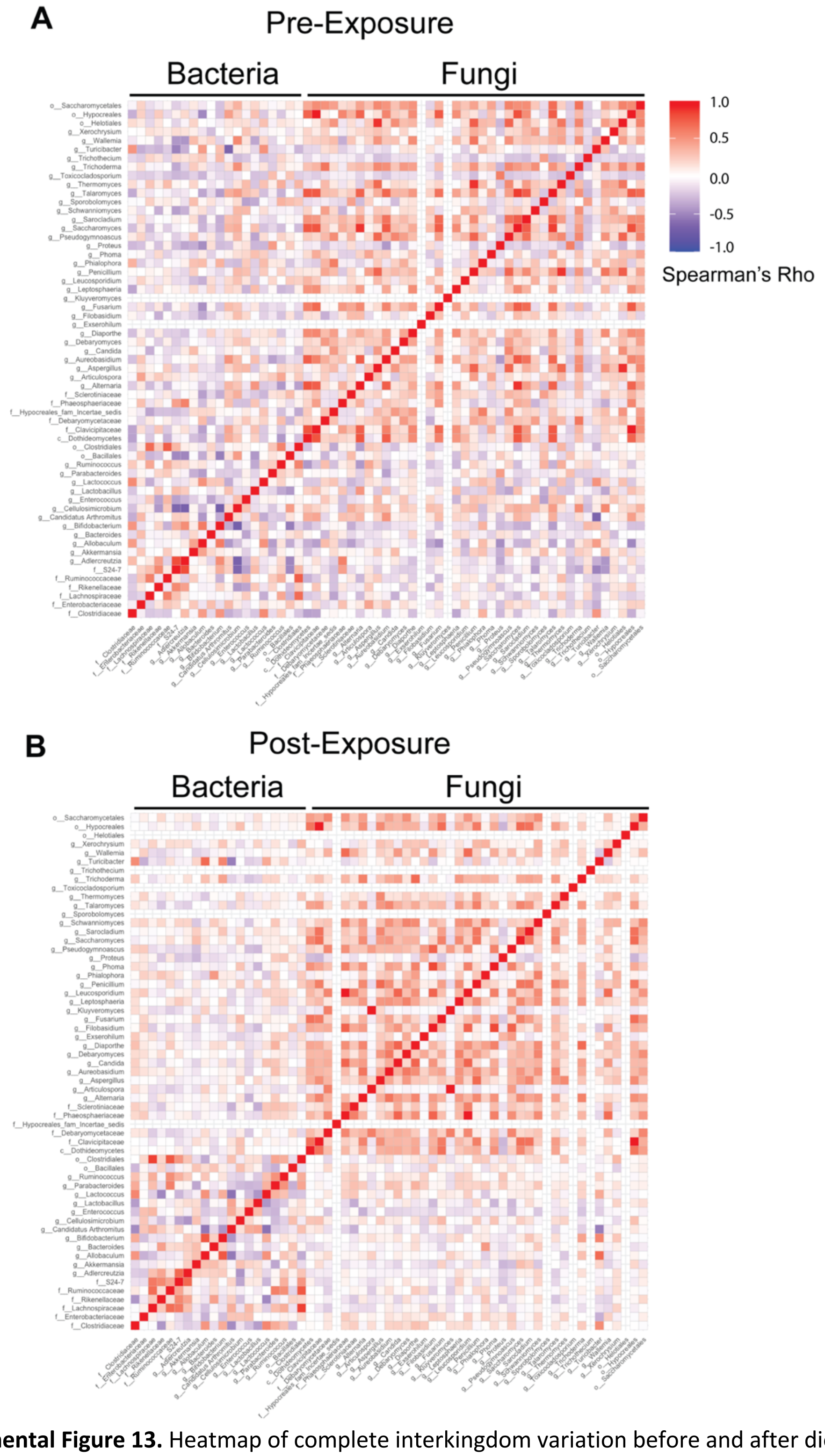

Supplemental Figure 13. Heatmap of complete interkingdom variation before and after dietary exposure. Interkingdom correlation analysis of bacterial and fungal taxa before and after exposure to processed diet in animals of both genders, shows a larger contribution of fungal correlated with differences in host metabolic outcomes than bacteria.
