# Supplementary material for: The gut mycobiome of healthy mice is shaped by the environment and correlates with metabolic outcomes in response to diet
Source: Commun Biol. 2021 Mar 5;4:281. doi: 10.1038/s42003-021-01820-z (PMC7935979; doi:10.1038/s42003-021-01820-z)
Supplement: Supplementary file 2 — Reporting Summary [file 42003_2021_1820_MOESM2_ESM.pdf]

nature research

Corresponding author(s): Joseph F. Pierre PhD
Kent A. Willis, MD

Last updated by author(s): 2/08/2021

Reporting Summary

Nature Research wishes to improve the reproducibility of the work that we publish. This form provides structure for consistency and transparency in reporting. For further information on Nature Research policies, see our Editorial Policies and the Editorial Policy Checklist.

Statistics

For all statistical analyses, confirm that the following items are present in the figure legend, table legend, main text, or Methods section.

n/a	Confirmed	
☐	☒	The exact sample size (*n*) for each experimental group/condition, given as a discrete number and unit of measurement
☐	☒	A statement on whether measurements were taken from distinct samples or whether the same sample was measured repeatedly
☐	☒	The statistical test(s) used AND whether they are one- or two-sided<br>*Only common tests should be described solely by name; describe more complex techniques in the Methods section.*
☐	☒	A description of all covariates tested
☐	☒	A description of any assumptions or corrections, such as tests of normality and adjustment for multiple comparisons
☐	☒	A full description of the statistical parameters including central tendency (e.g. means) or other basic estimates (e.g. regression coefficient) AND variation (e.g. standard deviation) or associated estimates of uncertainty (e.g. confidence intervals)
☐	☒	For null hypothesis testing, the test statistic (e.g. *F*, *t*, *r*) with confidence intervals, effect sizes, degrees of freedom and *P* value noted<br>*Give P values as exact values whenever suitable.*
☒	☐	For Bayesian analysis, information on the choice of priors and Markov chain Monte Carlo settings
☐	☒	For hierarchical and complex designs, identification of the appropriate level for tests and full reporting of outcomes
☒	☐	Estimates of effect sizes (e.g. Cohen's *d*, Pearson's *r*), indicating how they were calculated

Our web collection on statistics for biologists contains articles on many of the points above.

Software and code

Policy information about availability of computer code

Data collection	Data were generated through the Environmental Sequencing Facility at Argonne National Laboratory in Lemont, IL.
Data analysis	Operational taxonomic unit, metadata tables, univariate analysis results and R code are available from github.com/WillisLungLab/Vendors_gut_mycobiome.

For manuscripts utilizing custom algorithms or software that are central to the research but not yet described in published literature, software must be made available to editors and reviewers. We strongly encourage code deposition in a community repository (e.g. GitHub). See the Nature Research guidelines for submitting code & software for further information.

Data

Policy information about availability of data

All manuscripts must include a data availability statement. This statement should provide the following information, where applicable:

- Accession codes, unique identifiers, or web links for publicly available datasets
- A list of figures that have associated raw data
- A description of any restrictions on data availability

Sequences are available via the National Center for Biotechnology Information Short Read Archive (BioProject PRJNA597168).

Field-specific reporting

Life sciences study design

All studies must disclose on these points even when the disclosure is negative.

Sample size	Sample sizes of 6/group were included from each of the four commercial vendors at baseline and on each diet (at endpoint).
Data exclusions	No data were excluded from analysis.
Replication	Sequencing analysis were replicated in each biological sample.
Randomization	Animals were randomized to diets and experimental endpoints.
Blinding	Machine learning and modeling was performed in a blinded unbiased approach.

Reporting for specific materials, systems and methods

We require information from authors about some types of materials, experimental systems and methods used in many studies. Here, indicate whether each material, system or method listed is relevant to your study. If you are not sure if a list item applies to your research, read the appropriate section before selecting a response.

Materials & experimental systems

n/a	Involved in the study	
☒	☐	Antibodies
☒	☐	Eukaryotic cell lines
☒	☐	Palaeontology and archaeology
☐	☒	Animals and other organisms
☒	☐	Human research participants
☒	☐	Clinical data
☒	☐	Dual use research of concern

Methods

n/a	Involved in the study	
☒	☐	ChIP-seq
☒	☐	Flow cytometry
☒	☐	MRI-based neuroimaging

Animals and other organisms

Policy information about studies involving animals; ARRIVE guidelines recommended for reporting animal research

Laboratory animals	Mice, C57BL/6, both sexes
Wild animals	N/A
Field-collected samples	N/A
Ethics oversight	All animal studies were approved by the Institutional Animal Care and Use Committee at the University of Tennessee Health Science Center (protocol # 17-089).

Note that full information on the approval of the study protocol must also be provided in the manuscript.
